# Directed Evolution Reveals the Binding Motif Preference of the LC8/DYNLL Hub Protein and Predicts Large Numbers of Novel Binders in the Human Proteome

**DOI:** 10.1371/journal.pone.0018818

**Published:** 2011-04-18

**Authors:** Péter Rapali, László Radnai, Dániel Süveges, Veronika Harmat, Ferenc Tölgyesi, Weixiao Y. Wahlgren, Gergely Katona, László Nyitray, Gábor Pál

**Affiliations:** 1 Department of Biochemistry, Eötvös Loránd University, Budapest, Hungary; 2 Institute of Chemistry, Eötvös Loránd University, Budapest, Hungary; 3 Protein Modeling Research Group, Hungarian Academy of Sciences, Eötvös Loránd University, Budapest, Hungary; 4 Institute of Biophysics and Radiation Biology, Semmelweis University, Budapest, Hungary; 5 Department of Chemistry, University of Gothenburg, Gothenburg, Sweden; Science and Technology Facilities Council, United Kingdom

## Abstract

LC8 dynein light chain (DYNLL) is a eukaryotic hub protein that is thought to function as a dimerization engine. Its interacting partners are involved in a wide range of cellular functions. In its dozens of hitherto identified binding partners DYNLL binds to a linear peptide segment. The known segments define a loosely characterized binding motif: [D/S]_-4_K_-3_X_-2_[T/V/I]_-1_Q_0_[T/V]_1_[D/E]_2_. The motifs are localized in disordered segments of the DYNLL-binding proteins and are often flanked by coiled coil or other potential dimerization domains. Based on a directed evolution approach, here we provide the first quantitative characterization of the binding preference of the DYNLL binding site. We displayed on M13 phage a naïve peptide library with seven fully randomized positions around a fixed, naturally conserved glutamine. The peptides were presented in a bivalent manner fused to a leucine zipper mimicking the natural dimer to dimer binding stoichiometry of DYNLL-partner complexes. The phage*-*selected consensus sequence V_-5_S_-4_R_-3_G_-2_T_-1_Q_0_T_1_E_2_ resembles the natural one, but is extended by an additional N-terminal valine, which increases the affinity of the monomeric peptide twentyfold. Leu-zipper dimerization increases the affinity into the subnanomolar range. By comparing crystal structures of an SRGTQTE-DYNLL and a dimeric VSRGTQTE-DYNLL complex we find that the affinity enhancing valine is accommodated in a binding pocket on DYNLL. Based on the *in vitro* evolved sequence pattern we predict a large number of novel DYNLL binding partners in the human proteome. Among these EML3, a microtubule-binding protein involved in mitosis contains an exact match of the phage-evolved consensus and binds to DYNLL with nanomolar affinity. These results significantly widen the scope of the human interactome around DYNLL and will certainly shed more light on the biological functions and organizing role of DYNLL in the human and other eukaryotic interactomes.

## Introduction

The LC8 dynein light chain (DYNLL in mammals) is a highly conserved 10 kDa protein that was originally described as a potential cargo binding adapter of both dynein and myosin 5a motor proteins and as an inhibitor of the neuronal NO-synthase enzyme (reviewed in [1]). Subsequent studies demonstrated that it binds to and regulates dozens of other proteins unrelated to the cytoskeletal motors. It is now considered to be a eukaryotic hub protein [2], [3]. Its known binding partners are involved in highly diverse cellular processes including apoptosis (BH3-only proapoptotic proteins, Bim and Bmf [4], [5]), DNA repair (e.g. P53BP1 [6]), transcriptional regulation (e.g. NRF-1 [7]), nuclear transport (e.g. Nup159 and Pak1 [8], [9]), viral infection (e.g. lyssavirus P protein [10]) or cancer development (e.g. Pak1 [11]). Several DYNLL binders function in the presynaptic cytomatrix (e.g. Bassoon [12]) and in the postsynaptic density (e.g. GKAP [13] and KIBRA [14]). By gene knockout and knockdown experiments DYNLL has been shown to be an essential protein in *Drosophila* and *C. elegans* [15], [16]. Vertebrate genomes contain two DYNLL paralogs DYNLL1 and DYNLL2 which share 93% sequence identity at the protein level. It is still controversial whether the two isoforms have disparate cellular functions. In some studies DYNLL1 and DYNLL2 were found to bind *in vivo* specifically to the dynein and myosin 5a complexes, respectively [4], [17], [18], but other studies do not support this view [19]. The two isoforms have identical *in vitro* binding characteristics to their interacting proteins studied so far [18], [20]

Apo- and ligand-bound structures, determined both by X-ray diffraction and NMR spectroscopic studies, revealed that DYNLL forms a homodimer possessing two identical ligand binding sites. In DYNLL a swapped β-sheet dimer interface is formed. In the complexes two 7-residue peptides bind to parallel grooves at the edges of the dimer interface expanding the two central β-sheets [18], [21], [22], [23], [24], [25], [26], [27]. Since the DYNLL-binding linear peptide motifs are generally located in intrinsically disordered regions of the partner proteins and bind to DYNLL as a short β-strand they could be considered as β-MoRE (molecular recognition element) [28], [29]. DYNLL-binding motifs are often flanked by coiled coil or other dimerization domains. DYNLL could function upon complex formation as a “molecular glue” by promoting and stabilizing the dimeric structure and in this way regulating the function of its binding partners [30], [31], [32].

The diverse DYNLL binding motifs were traditionally divided into two loose consensus sequence classes, [K/R]_-3_X_-2_T_-1_Q_0_T_1_ and G_-2_I_-1_Q_0_V_1_D_2_ [33], [34]. Moreover, a few known partners have non-canonical motifs lacking the conserved Gln such as myosin 5a (D_-5_D_-4_K_-3_N_-2_T_-1_M_0_T_1_D_2_) [30], [32], p21-activated kinase (Pak1) (R_-5_D_-4_V_-3_A_-2_T_-1_S_0_P_1_I_2_) [35] and GRINL1 (E_-5_T_-4_R_-3_E_-2_I_-1_G_0_V_1_G_2_) [36]. In the crystal structure of canonical complexes the side chain of the central glutamine (position 0) caps the N-terminal end of the second α-helix, while the side chains of residues at positions +1, −1 and −3 interact with the hydrophobic interior of the binding groove and some of these also participate in H-bonds formed by structural water molecules [22], [23], [24], [26]. The importance in binding of the conserved glutamine and of the two flanking residues was demonstrated by mutational studies [37]. In the non-canonical Pak1 sequence, a key H-bond network was identified that compensates for the lack of the conserved glutamine [35]. The functional significance of the binding motif classes has not been demonstrated yet. Previous studies suggest a hierarchy or continuum of binding affinities (∼1–50 µM) depending on the actual sequence of the motifs [20], [35]. On the other hand, different motif classes could achieve roughly the same binding affinities through different thermodynamic mechanisms [20]. It is important to note that many of the binding partners were shown to exist as homodimers therefore DYNLL most likely forms dimer-dimer complexes with its partners [24], [30], [35]. In fact, binding of bivalent ligands shows a considerable avidity effect; affinity of a dimeric myosin 5a fragment has been recently reported to be ∼40 nM [20].

In this work we applied phage display in order to determine the characteristic amino acid preferences of individual peptide motif positions. Seven positions in a naïve peptide-phage library were totally randomized, while the conserved glutamine was fixed. The *in vitro* selected consensus sequence is similar to the natural one, but is extended by an additional binding determinant, a Val, which increases the affinity twentyfold. Dimerization through a Leu-zipper further increases the affinity into the subnanomolar range. Structural basis of the affinity enhancement was addressed by solving the crystal structure of two DYNLL-peptide complexes. Using the *in vitro* evolved sequence pattern we performed a bioinformatics analysis to identify potential novel DYNLL-binding proteins. By focusing our search on intrinsically disordered regions of intracellular segments of the human proteome we identified 110 potential novel DYNLL binding partners. Interestingly, among several promising candidates, we identified a human protein, EML3 (also termed as EMAP-3, Uniprot: Q32P44) that contains the phage-selected consensus sequence located in a disordered region. With *in vitro* studies we verified that EML3 binds to DYNLL with high affinity.

## Results

### Directed evolution of DYNLL-binding motif from bivalently displayed naïve peptide library

We combined all 41 hitherto identified canonical DYNLL-binding motifs representing both previously established classes from 33 eukaryotic interacting proteins (Table S1) to define the following 7-mer linear motif: ([D/S]_-4_K_-3_X_-2_[T/V/I]_-1_Q_0_[T/V]_1_[D/E]_2_. Published affinities of the natural binding peptides are in the micromolar range (0.75 µM<K_d_<50 µM) [20], [38], [39].

**Figure 1:**
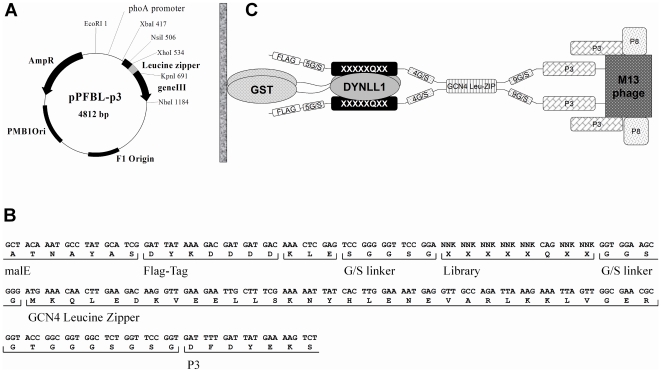
Bivalent phage display.

We aimed to elucidate the thermodynamically driven characteristic amino acid preferences of individual motif positions. To do so, we applied directed evolution by selecting DYNLL1 binders from a phage displayed peptide library. Before building the library we determined the optimal display format by displaying the strongest known binding peptide (K_d_ = 0.75 µM) from Bmf [20] on M13 phage. The Bmf peptide (E_-5_D_-4_K_-3_A_-2_T_-1_Q_0_T_1_L_2_) was fused to an N-terminal Flag-tag and displayed monovalently on the p3 and also multivalently on the p8 coat protein. Phage-ELISA using immobilized anti Flag-tag antibody demonstrated successful display in both cases. However, binding to immobilized GST-fused DYNLL1 was hardly detectable (data not shown). DYNLL isoforms are homodimers having two identical peptide binding sites [22], [23], [24], [26]. In order to mimic the natural dimer binding to dimer mechanism [20], [24], [30], [40], we inserted a Leu-zipper motif from GCN4 between the Flag-tagged Bmf peptide and the p3 coat protein and displayed the peptide in a bivalent format (see Figure 1). A similar dimerizing approach was published by Fuh and colleagues [41],[42]. In that study the authors applied competition phage-binding assays and demonstrated the bivalent display through its apparent affinity boosting avidity effect that, as expected, occurred only on surface immobilized targets as opposed to solvent-phase targets. In our case, due to the same effect, Leu-zipper based dimerization resulted in detectable binding to GST-fused DYNLL1. Based on simple modeling studies we produced three variants with 4-mer (GGSG), 6-mer (GGSGGS) and 8-mer (GGSGGSGS) linker, respectively, in order to optimize the geometry of the bivalent binding. In binding assays the 4-mer linker was the best one. Since both too low or too high transcription levels decrease display efficiency [41], [43], we tried to optimize the expression level of the originally used pTac promoter by inducing it at various IPTG concentrations. As low as 10 µM IPTG concentration had deleterious effect on display (Figure S1) suggesting that even the leakage of the strong pTac promoter might afford higher than optimal transcription level. Therefore we replaced the pTac promoter with the weaker PhoA promoter [42], which increased the display level about 30-fold (Figure S1) allowing for efficient peptide-library display.

The library design fixed the most conserved glutamine at position 0, while in all other positions from −5 to 2 allowed the occurrence of all 20 amino acid residues: X_-5_X_-4_X_-3_X_-2_X_-1_Q_0_X_1_X_2_ (Figure 1). Note that we extended the original motif with one extra position (position -5) at the N-terminus. The library contained 2.1×10^10^ individual clones. Total randomization of 7 amino acid positions by degenerate (NNK) codons results in a theoretical diversity of 32^7^ = 3.4×10^10^ variants. Our library size approaches the theoretical one suggesting that most variants were indeed present in the starting repertoire. In the third panning cycles the enrichment was 100 fold. Individual clones from this selection cycle were tested in phage-ELISA experiments for DYNLL1-binding and the DNA of 36 positive clones was sequenced yielding 25 unique sequences (Table S2). From the anti Flag-tag selection 32 ELISA positive clones were sequenced and 30 were unique (Table S3). This set was used for subtracting the effects of display bias from the DYNLL1-selected set.

### The *in vitro* evolved binding motif

**Figure 2:**
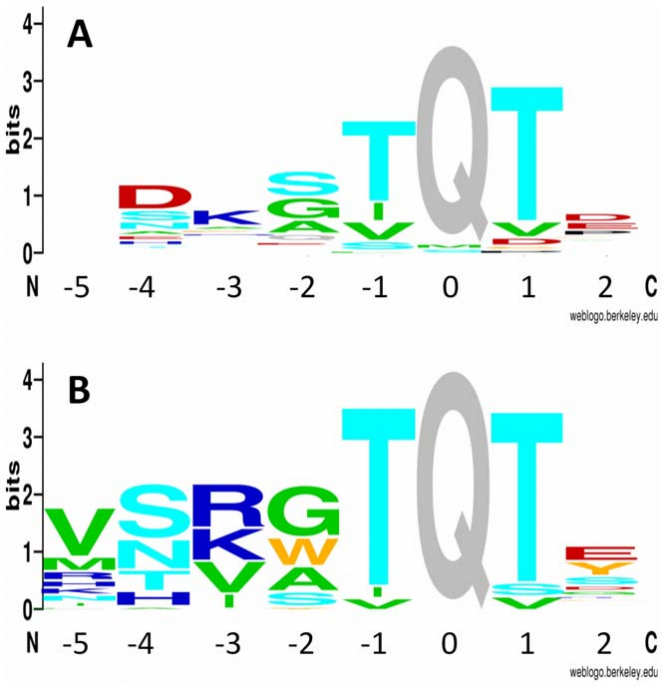
Sequence logos of naturally evolved and *in vitro* evolved binding motifs. Position heights represent the degree of conservation. Letter heights indicate normalized amino acid proportions. (A) Sequence logo calculated from 41 known natural binding motifs listed in Table S1 and (B) from phage selected DYNLL-binding clones listed in Table S2. Similar colors indicate similar chemical properties [44].

After display-bias normalization we illustrated the amino acid preferences of individual binding positions in the form of a sequence logo using the WebLogo program [44]. In Figure 2 the logo of the *in vitro* evolved pattern is compared to the logo of 41 known naturally evolved DYNNL1 binding peptides (listed in Table S2). Generally, the two logos are similar, but there are a few characteristic differences. In the −1 and +1 positions both logos show strong conservation and marked preference for threonine. At position +2 there is a lower level conservation and slight preference for a negative charge (Glu, Asp) in both sets. There are similarities at the moderately conserved positions −2 and −3 as well. At position −2 mostly small residues are selected (Gly, Ala), while at position −3 there is a preference for positively charged residues (Arg, Lys). The characteristic differences appear at positions −4 and −5. In the natural binding peptides at position −4 a negatively charged Asp is the most frequent amino acid, which is absent from the *in vitro* selected pool. The most striking difference emerged at position −5, where the known natural binding peptides show lack of preference, while the *in vitro* evolved set presents significant non-randomness. Here mostly apolar residues (Val, Met, Ile, Leu) or polar residues with long aliphatic side chain portion were selected (Arg, Lys), with the most preferred residue being Val.

### Binding properties of the *in vitro* evolved consensus peptide − the significance of Val_-5_

**Figure 3:**
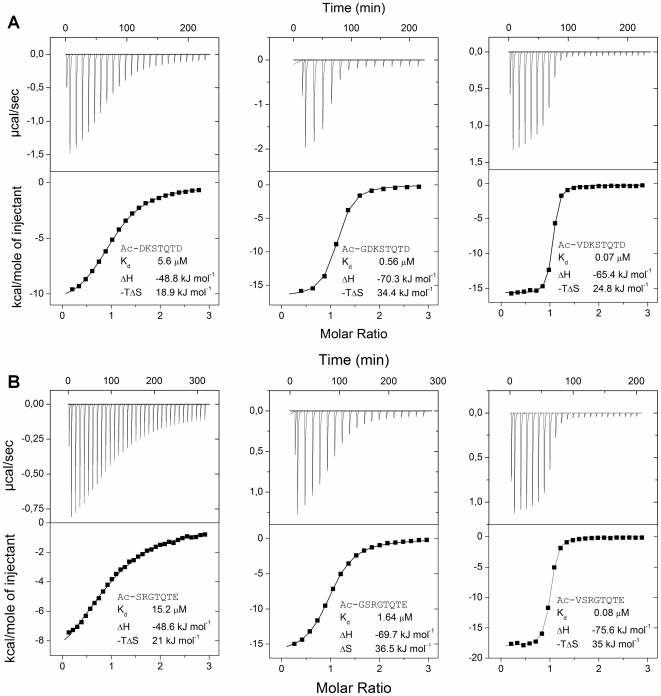
Thermodynamic binding properties of natural and *in vitro* evolved motifs determined by isothermal titration calorimetry. Data for natural consensus (Ac-DKSTQTD) and its two control peptides and data for phage selected consensus (Ac-VSRGTQTE) and its two control peptides are shown in A and B, respectively.

Binding parameters of the *in vitro* evolved Ac-VSRGTQTE and naturally evolved Ac-DKSTQTD consensus peptides were determined by isothermal titration calorimetry (ITC). Synthetic peptides were used and N-terminal acetylation was applied to better mimic a non-terminal peptide motif by avoiding the positively charged N-terminus. In order to elucidate the particular contribution of Val_-5_ to the binding affinity, several control peptides were produced. The *in vitro* evolved Val_-5_ was replaced with a glycine (Gly_-5_) to assess side chain function. In a further truncated variant the entire amino acid residue was replaced with an acetate group (Ac_-5_) to assess the function of the amide group at position −5. The same variations of Val_-5_, Gly_-5_ or Ac_-5_ were also introduced in the framework of the naturally evolved consensus peptide. DYNNL1 binding of all six variants was analyzed by ITC. The results are illustrated in Figure 3 and the binding parameters are summarized in Table 1. In all measurements the stoichiometry of binding was 1∶1 (2 peptides binding to one homodimer DYNNL1). The binding was enthalpy-driven and was accompanied with unfavorable entropy change. Ac_-5_ versions of both the natural and the *in vitro* evolved consensus peptide had binding affinities in the micromolar range. Replacing the acetyl group with Gly_-5_ led to a tenfold affinity increase with both peptides. This was due to a favorable change in binding enthalpy contribution suggesting that the main chain at position −5 participates in stabilizing molecular interactions. Replacing Gly_-5_ with a Val increased the binding affinities even further and at least in the frame of the *in vitro* evolved peptide it was due to a favorable binding enthalpy change suggesting new stabilizing interactions through the Val side chain.

**Table 1:** Thermodynamic analysis of DYNLL-binding interactions.

Partner	*K_d_* (µM)	Δ*H* (kJ mol^−1^)	*-T*Δ*S* (kJ mol^−1^)
Ac-DKSTQTD	5.6	−48.8	18.9
Ac-GDKSTQTD	0.56	−70.3	34.4
Ac-VDKSTQTD	0.07	−65.4	24.8
dimeric-GSGDKSTQTD*	<0.007	−76.2	<29.4
dimeric-GSVDKSTQTD*	<0.009	−88	<42.0
Ac-SRGTQTE	15.2	−48.6	21.0
Ac-GSRGTQTE	1.64	−69.7	36.5
Ac-VSRGTQTE	0.08	−75.6	35.0
dimeric-GSGSRGTQTE*	<0.003	−69.6	<20.6
dimeric-GSVSRGTQTE*	<0.007	−64.9	<17.6
EML3 (8-94)	0.05	−32.4	9.465

*Data reach the dynamic range limit of the ITC method.

### Peptide dimerization increases binding affinity

Homodimer structure of DYNLL1 allows for simultaneous binding of two peptides resulting in an avidity effect provided that the peptides are presented in dimer format [20], [24]. This effect was utilized in the *in vitro* evolution process by displaying the peptide library in a Leu-zipper fused bivalent form. We tested how the same bivalency affects binding properties of the peptides. Dimerization of the Gly_-5_ containing peptides enhanced their affinity compared to the monomeric versions (see in Table 1). In case of the naturally evolved consensus the improvement was 80-fold, while for the *in vitro* evolved consensus it was over 500-fold. Interestingly, for the *in vitro* evolved peptide this improvement was solely due to a less unfavorable entropy term, suggesting that pre-dimerization of the peptide decreases the entropy penalty of complex formation. In case of the naturally evolved consensus peptide the affinity improvement was due to a combination of more favorable enthalpy and less unfavorable entropy terms. In both cases the affinities were driven to the low nanomolar range, where ITC measurements reach the dynamic range limit of the method [45] suggesting that affinities of the dimeric peptides can be even higher. Dimerization of the Val_-5_ containing naturally evolved and *in vitro* evolved peptides resulted in a tenfold improvement of their monomeric affinities resulting similar low nanomolar values already seen for the Gly_-5_ peptides. Again, the corresponding dissociation constants might be significantly lower.

### Structural significance of position −5

For crystallographic studies we used DYNLL2 because its binding site and binding properties are identical to those of DYNLL1 [20], but it lacks a free surface exposed cysteine and hence is chemically more stable during crystallization. In order to understand the positive contribution of Val_-5_ to DYNLL binding we crystallized and analyzed the DYNLL2 complexes formed with the monomeric Ac-SRGTQTE peptide and with the Leu-zipper dimerized GSVSRGTQTE peptide (where the first two residues originate from the recombinant construct) (Table S4). We also made multiple attempts to grow crystals of acceptable quality from the Ac-VSRGTQTE peptide-DYNLL2 complex. Initially, this complex was prepared similarly to the Ac-SRGTQTE peptide-DYNLL2 complex (see Experimental Procedures), but it showed markedly different behavior. After 5 minutes thousands of tiny crystal groups composed of dozens of small needles appeared. Several thousands of conditions were tested, but even the best crystals diffracted only around 10 Å.

**Figure 4:**
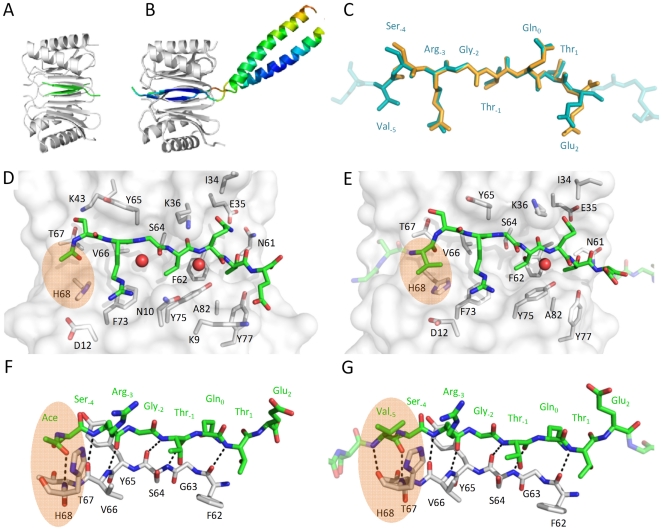
Crystal structure of Ac-SRGTQTE – DYNLL2 (A, C, D and F) and Leu-zipper dimerized VSRGTQTE – DYNLL2 complex (B, C, E and G). Peptides bound to DYNLL adopt similar conformation, see alignment in C. As shown in B the Leu-zipper dimerized VSRGTQTE peptides lay into two parallel binding grooves on DYNLL2 forming a dimer-dimer complex. Colors from red to blue correspond to high and low B-factors, respectively (from 131 to 48). The GGSG linker between the binding motif and the Leu-zipper appears to be flexible. Side chain interactions between the peptides and the binding grove of DYNLL2 are shown in D and E. Side chains of DYNLL2 having at least one atom within 4 Å distance from the peptide are shown as sticks. F and G are rotated versions (by approximately 90° along the axes of the binding motifs) of D and E, respectively, and show that the peptides bind to DYNLL2 in a β-strand conformation. Interestingly, the interaction is partially mediated by H-bridges of several buried structural waters represented as red spheres in D and E. Comparing F and G shows that the backbone of Val_-5_ forms one additional hydrogen bond, while E and G illustrate that its side chain interacts with DYNLL2 His68. Details of this interaction are highlighted by orange areas.

Crystal structure of the Ac-SRGTQTE-DYNLL2 complex was solved and refined at 1.31 Å resolution (Figure 4A). The asymmetric unit contains four chains forming two dimers. In the resulting electron density map the bound Ac-SRGTQTE-peptide could be clearly identified. All residues were in the most favored Ramachandran region except for Asn51, which has a positive ϕ angle, as in all other DYNLL structures [18], [21], [22], [23], [24], [25], [26], [27]. Similar to the human DYNLL/Swa peptide complex (PDB entry 3e2b), each dimer contains two five-stranded β sheets, with four strands from one subunit and a fifth strand crossed over from the other subunit. One side of each sheet is flanked by two helices while the other side forms the dimer interface. Both Ac-SRGTQTE peptides bind identically to their symmetry related binding sites forming a sixth β strand in the grooves created at the dimer interface (Figure 4F). The side chains of residues Thr_1_, Thr_-1_ and Arg_3_ of the peptide project toward the interior of DYNLL2, into a deep hydrophobic groove lined by aromatic DYNLL residues Phe73, Tyr75, Tyr77, Phe62 and Tyr65 (Figure 4D).

Crystal structure of the complex formed between DYNLL2 and Leu-zipper dimerized GSVSRGTQTE peptide complex was solved and refined to 2.9 Å resolution (Figure 4B). Overall conformation of the DYNLL2 dimer and the binding region highly resembles to that of the Ac-SRGTQTE complex structure. Individual DYNLL2 dimers can be fitted with an RMSD in the range of 0.38–0.66 Å for the DYNLL2 backbone atoms and with an RMSD in the range of 0.42–0.61 Å for the peptide (Figure 4C). A few side chains outside of the motif segment were not resolved in the electron density map perhaps due to inherent flexibility. The binding region was well resolved due to conformational stabilization by molecular contacts. The DYNLL2 dimer and the interactions within the two binding grooves are symmetric. In contrast, the coiled coil region of the Leu-zipper diverges from the symmetry axis of the complex due to asymmetric crystal contacts of its two α-helices. Different backbone conformations of the four-residue linkers in the two peptides as well as their elevated thermal factors indicate that the linkers are flexible (Figure 4B) suggesting that the Leu-zipper does not interfere with the binding interaction. The bound SRGTQTE segment of the peptide preserves the 5 backbone hydrogen bonds of the β-sheet interaction and the 11 additional hydrogen bonds of the high resolution complex structure (Figure 4F). Favorable interactions of the additional Val_-5_ residue highlight direct and indirect structural basis of its contribution to increased binding affinity. The Val_-5_ residue extends the β-sheet establishing two backbone hydrogen bonds (Figure 4G), while its side chain is accommodated into a shallow pocket formed partially by the His68 imidazole ring, which is also involved in binding the Arg_-3_ side chain through an H-bond (Figure 4E). The recombinant construct derived Gly_-7_ and Ser_-6_ residues do not interact directly with DYNLL2.

In the crystal of the dimer-to-dimer complex, due to crystal packing, neighboring GSVSRGTQTE-DYNLL2 units form antiparallel β-sheet structures. We speculate that the same antiparallel β-sheet structures form in case of the Ac-VSRGTQTE-DYNLL2 complex resulting in the observed almost instantaneous crystal formation. If this is the case, presence of the Leu-zippers would slow down the association of the resulting long linear “rods”, and that is why crystallization of dimerized GSVSRGTQTE proceeds at a much lower rate.

### Predicting novel DYNNL binding partners based on the phage-selected sequence set

**Figure 5:**
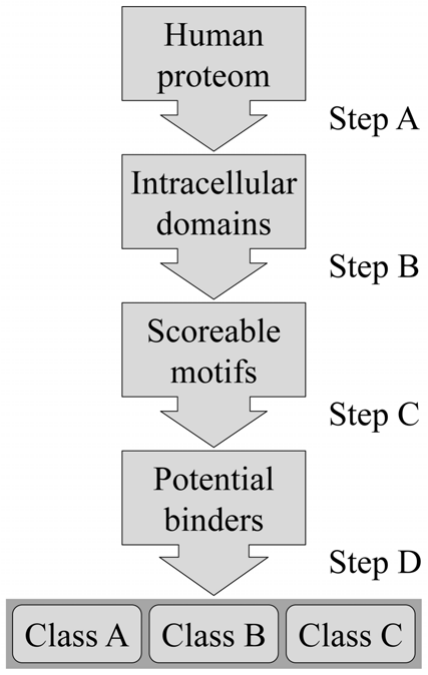
Flowchart of the binding partner prediction. Step A: all non intracellular segments were excluded from the search. Step B: sequences were split into overlapping eight residue segments; only disordered segments with Gln at position 0 were scored. Step C: a score was assigned (see Methods). Motifs with scores above threshold are considered potential DYNLL binders. Step D: based on amino acid composition, motifs were sorted into three classes with different binding probabilities.

It was previously shown that display bias-normalized affinity-selected amino acid frequencies correlate with binding energy contributions of the selected amino acid residues [46], [47], [48], [49], [50], [51]. This finding together with the assumption that amino acids in a linear motif contribute independently to the binding affinity, led to the design of a simple scoring system (see Materials and Methods). The general logic of binding motif prediction is illustrated as a flowchart in Figure 5. When an octapeptide segment is aligned with the phage-selected sequence logo, at each position the given amino acid type of the peptide gets scored based on the display-bias normalized frequency of the same amino acid type in the selected pool. The eight positional score values are summed up to give the final score (see Materials and methods). This system is suitable for the 7 fully randomized positions but is incompatible with the fixed Gln at position zero. Giving zero score at this position for the lack of a Gln would have underestimated such motifs compromising the entire scoring system. Therefore we analyzed only those segments that do have a Gln at position zero and we did not give a score to this position. This way the minimal score, zero is given to octapeptides that out of the fixed Gln do not contain any residue present in the selected set. The maximal score, 367 is given to the octapeptide representing the consensus of the selected pool.

**Figure 6:**
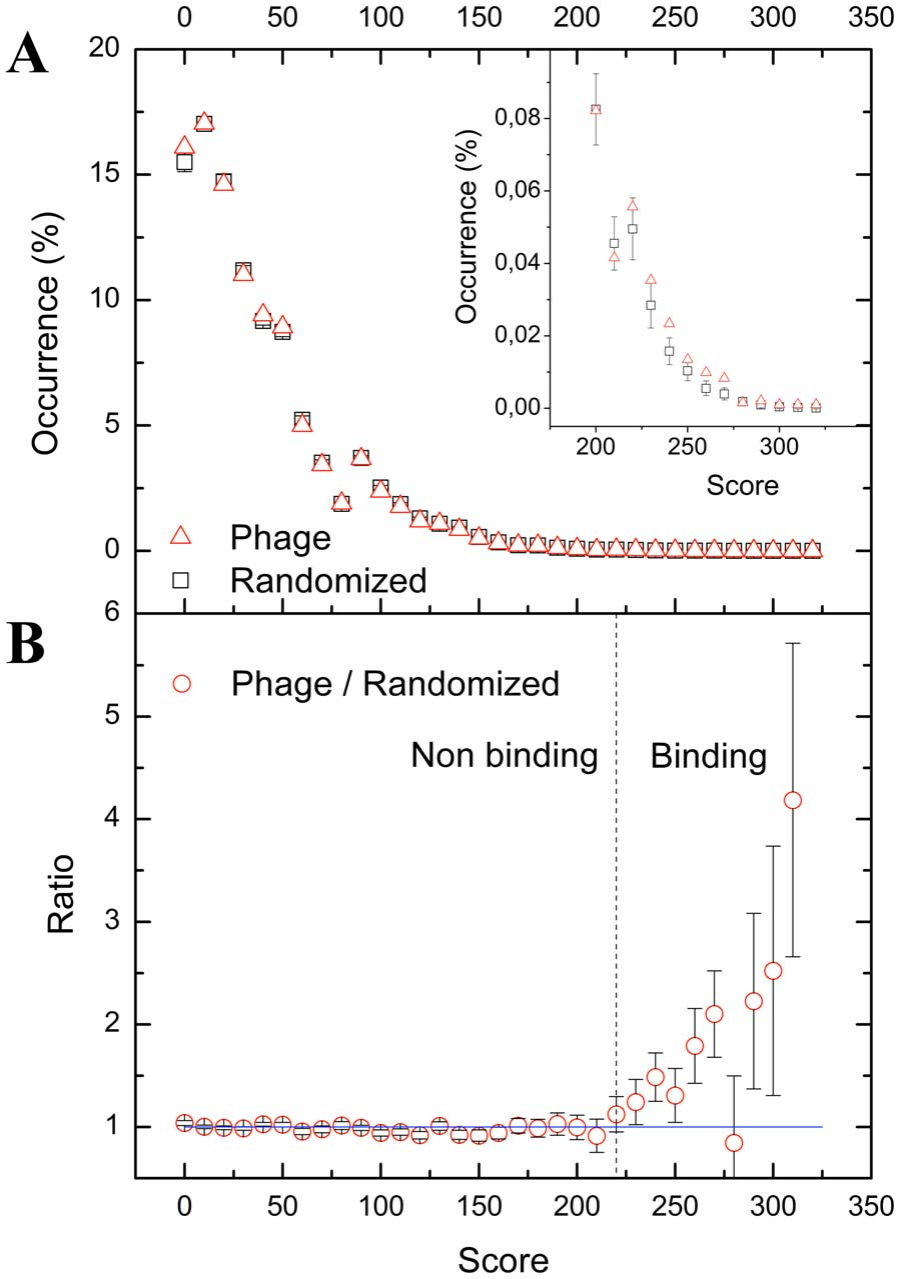
Statistical analysis for threshold score determination. (A) Density distributions of scores. Dashed baseline is derived from average values from 1000 randomized score sets (see Methods) while continuous line represents the *in vitro* selected scores. In the high score region (inset), the phage-selected set yields higher frequencies than the randomized baseline set. (Error bars show standard deviation of frequencies in the randomized sets). (B) The ratio of the density distribution functions of the scores. Above a threshold score value of 220 predicted DYNLL-binding motif frequencies exceed stochastic frequencies obtained from the randomized set, therefore their ratio exceeds one.

We restricted our search to cytoplasmic and predictably disordered regions of the human proteome since known DYNLL binding motifs are limited to these sequence classes. The density distribution of the scores from 0 to 367 shows a nearly continuous, exponentially decaying profile (Figure 6A.). The score distribution of known binding motifs (Table S1) shows that: i) the two motif families, [K/R]_-3_X_-2_T_-1_Q_0_T_1_ and G_-2_I_-1_Q_0_V_1_D_2_ are separated from each other, the former having higher scores suggesting that members of this family are closer to the thermodynamic binding optimum; and ii) members of the [K/R]_-3_X_-2_T_-1_Q_0_T_1_ family are dispersed in the high score region. Considering these as inner controls their distribution indicates that the scoring system is appropriate to predict novel partners.

A fundamental question was how to set the cutoff score value in order to define a set of predicted novel binders depleted in false positives. The global decaying trend of the probability density function of scores is dictated by simple statistical rules. Starting with an arbitrary sequence pattern for which no binding protein exists in the human proteome the same global trend of scores would apply. We argue that in case of a sequence pattern for which a corresponding binding protein does exist, natural selection should drift the proportion of high-score motifs away from the stochastically expected values. Following this logic we used a bootstrap analysis to determine the statistically driven baseline of the probability distribution as follows. We produced one thousand different scoring matrices where the positional amino acid frequencies were kept the same as in the real phage-selected matrix, but the positions were scrambled. Out of these thousand scrambled patterns only a few if any might coincidentally represent preference of an existing protein binding site and that is why these can be used as a control set. We divided the density values obtained from the DYNLL scoring with the corresponding mean values from the scrambled scoring analysis. Below a score value of 220 the normalized density values were very close to one, but above that score a statistically significant amount of high-score motifs was detected (Figure 6B). Thus, we used the score value of 220 as a statistically verified cutoff to define our predicted dataset of DYNLL binders. Proteins with motif(s) having a score 220 or higher are considered to be potential DYNLL binders. Normalized density values above score 220 indicates positive natural selection that evolved a large number of DYNLL-binding partners, which is expected for a hub protein.

Above the threshold level there are 242 motifs provided by 219 human proteins (listed in Table S5). Among these we find 8 out of the 18 human DYNLL binding motifs for which the binding site had already been identified. Interestingly, the highest possible score i.e. the phage-selected consensus sequence is present once in the human proteome in a microtubule associated protein called EML3 [52], which has not yet been identified as a DYNLL interacting partner. Our preliminary ITC studies verified that a recombinant fragment of EML3 (residues 8–94) containing a coiled coil domain and the *in vitro* evolved consensus motif binds to DYNLL with a K_d_ of 50 nM (Figure S2).

## Discussion

Most biological processes manifest through complex networks of protein-protein interactions. In order to understand how a network functions, one needs to identify all individual interactions and determine their affinity and specificity. DYNLLs are hub proteins recognizing a short linear motif, a β-MoRE in their growing number of newly identified binding partners [2], [37].

As residue conservation indicates functional importance, comparison of natural binder sequences could in principle elucidate the preference of their target binding site. However, known natural sequences usually represent only a subset of all existing binders biasing the observed sequence trend. Moreover, natural evolution can select sequences based on several functions in addition to binding, which complicates interpretation of an observed pattern. A systematic study of the binding epitope through single amino acid replacements can test the functional importance of individual residues, but a complete analysis of even a small binding site would require an impractically large number of mutants. If the function relies on cooperating residues, mutation combinations might require astronomical number of variants. Directed evolution combines combinatorial mutagenesis with functional selection and easily handles the above problems [53]. Diversity of our phage display library covered almost all possible mutation combinations representing a near complete, unbiased starting variant set. Our *in vitro* binding-selection combined with a powerful display bias normalization revealed characteristic sequence features, but we have to keep in mind that sequence patterns obtained this way are the results of a thermodynamics-driven binding-selection as opposed to a complex natural selection. Based on the phage-selected binding pattern we applied a simple scoring system combined with an untraditional baseline correction approach to identify novel natural binding partners in the human proteome.

Our library design covered both natural DYNLL-binding sequence classes. Importantly, the 25 unique phage-selected binders demonstrated that the two class characteristics can freely mix. Thus, the existence of the two natural classes is not due to a simple binding selection. It is either due to functional selections other than DYNLL binding, or could simply be a sampling error due to the limited number of known natural DYNLL binders. The same conclusion was drawn from the *in vitro* thermodynamic characterization of target sequences binding to DYNLL [20].

ITC measurements show that dimerization of the peptides leads to an at least 100 fold increase in binding affinity. For successful phage selection it was necessary to start with a bivalently displayed peptide library. Nevertheless, the resulted sequence pattern is relevant for the monovalent interaction as well. Monomeric form of the consensus binding motif (VSRGTQTE, K_d_ = 0.08 µM) binds tenfold tighter than the strongest known natural DYNLL-binding sequence of the Bmf protein (EDKATQTL, K_d_ = 0.75 µM) [20]. The natural and the *in vitro* evolved sequence patterns had strong similarities at positions −1 and +1 localizing the TQT segment as a binding epicenter. Additional similarities in preferences were found at positions −3, −2 and +2 suggesting that natural selection at these positions is also governed mostly by DYNLL-binding. However, compared to the TQT motif there is a relatively low level of conservation at these positions in both sets indicating less stringent requirement for a particular stereochemistry.

We identified two positions with characteristic differences between naturally and *in vitro* evolved preferences. In nature Asp is the most frequent residue at position−4, but it is absent from the *in vitro* selected population, where the second most frequent residue in nature, Ser was selected. The anti Flag-tag antibody selected clone set showed that peptides having Asp within the octamer segment can be readily produced and displayed on phage. Therefore lack of Asp_-4_ in the binding selected population is due to a negative selection suggesting that in nature Asp_-4_ might have a functional role other than DYNLL-binding. This model is complicated by the fact that both in the nNOS-DYNLL [22] as well as in the Pak1-DYNLL complex [35] Asp_-4_ appears to contribute to the binding energy through H-bonding to Thr67 of DYNLL. Interestingly, in the *in vitro* evolution derived Ac-SRGTQTE-DYNLL2 complex Ser_-4_ participates in an analogous H-bond with the same threonine.

The perhaps most important finding was that upon *in vitro* evolution position −5 became much more conserved than observed in nature. This suggested that position −5 could increase DYNLL-binding affinity, but at least by the hitherto identified set of natural DYNLL-binders this capacity is not generally utilized. Indeed, ITC measurements verified that the most abundant *in vitro* selected residue at this position, Val_-5_ significantly increases binding affinity. Both the structure of the Leu-zipper dimerized VSRGTQTE peptide - DYNLL2 complex as well as modeling studies (unpublished results) showed that the apolar side chain of Val_-5_ packs against His68 of DYNLL2, while the Val_-5_ main chain forms two H-bonds with the protein further stabilizing the antiparallel β-sheet structure of the complex. Our findings on the functional role of Val_-5_ are in good agreement with the pepscan analysis of Lajoix and colleagues [37] who found that a Val or Ile at position −5 contributes to binding affinity.

One trivial explanation for nature rarely utilizing binding potential of position −5 can be that natural evolution optimized this interaction to have moderate affinity providing a dynamic, transient nature. Although this might be the general rule, we already identified one human protein, EML3 that contains a perfect match of the *in vitro* evolved consensus. The sequence of the DYNLL-binding motif is predicted to be located in a disordered local environment with a nearby dimerizing coiled-coil segment. These features increase the likelihood that EML3 is a novel DYNLL binding protein. If so, EML3 is expected to bind DYNLL with unusually high affinity suggesting that their interaction is less transient than those so far identified. We have already shown that a large fragment of EML3 binds tightly to DYNLL and studies are in progress to further characterize the DYNLL-EML3 interaction and to determine its possible function.

Based on the phage selection results we performed a bioinformatic analysis on the human proteome to filter out a set of potentially novel DYNLL binders. Had we aimed to cover all known motifs we would have contaminated the filtered set with a large number of false positives. Instead, we aimed to produce a high-probability set with minimal amount of false positives even if we would exclude several known DYNLL-binders (false negatives). By applying a normalized scoring system we identified over two hundred potential novel DYNLL binders. On the score density function plot these exceed a statistically established threshold suggesting that this set should contain few false negatives. Moreover, novel members of this set are interspersed with known natural binders, which validate our analyses (Table S1). On the other hand, known binding partners getting lower than threshold scores are “false negative” in this test. These low scores indicate that factors other than thermodynamic adequacy of the octamer motif can facilitate DYNLL binding. We have already demonstrated that a “dimer binding to dimer” scenario providing avidity is such a factor, which can increase apparent affinity by several orders of magnitude [20]. Similar effect is expected when DYNLL binding occurs in the context of a multimeric protein complex. Extending the prediction to such cases requires more accurate protein annotation and further experiments. For example, although the coiled coil motif is the most studied predictable dimerization engine and many DYNLL binders contain a nearby coiled coil segment, it is yet to be elucidated how binding affinity depends on the distance between a DYNLL binding site and the coiled coil.

**Figure 7:**
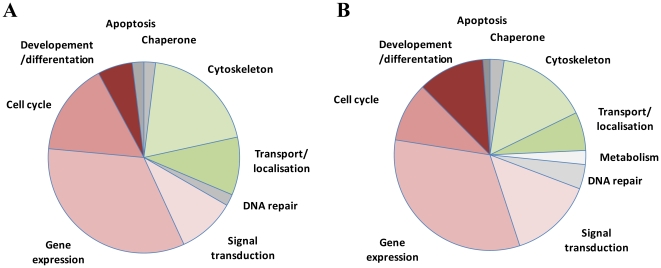
Functional distribution of 41 known (A) and 110 predicted (B) binding partners of DYNLL. Data were collected from the functional and Gene Ontology annotations of UniProt entry for each protein.

Each positional score reflects the frequency of the corresponding amino acid at that position in the phage selected sequence set therefore its value cannot be negative. This way the same zero score is given for the absence of an amino acid irrespectively whether it is missing for being energetically inert, or for being inhibitory. This latter case should be given a negative penalty score, but that is incompatible with our phage selection approach. As an alternative solution we checked all cases where a predicted motif position was given a zero score. We argued that if the residue missing from the phage selected set exists at the same position in known DYNLL binders, than it should be inert rather than inhibitory. Moreover, based on the result of the pepscan study of Lajoix *et al* [37] one residue type, proline is highly inhibitory, in core positions (-4^th^ – 0^th^) it disrupts binding. In line with the above arguments we sorted the predicted high score motifs into three subclasses in descending order of reliability (Table S5): The most likely binders, 110 binding motifs contributed by 98 proteins, belong to class A where all residues of the motifs are present either in the phage selected pool or in known DYNLL binding motifs suggesting no energetic obstacles for binding. The 84 predicted motifs belonging to 80 proteins in class B contain up to three positions where the residue is missing from both the phage selected and the known natural binding motif set. In class C containing 49 motifs from 49 proteins the presence of proline in core positions may disrupt binding, like in the case of myosin 5a, where an alternatively spliced form contains a proline in the -4^th^ motif position, which renders the binding undetectable [30]. Although these sequences are less likely to bind, favorable sequential environment resulting in high overall score together with the ability of multimerization may help to overcome the impairing effect of prolines in some of these predicted motifs. We compared the functional profile of the class A predicted pool to that of known DYNLL binders (Figure 7) and found striking similarities. Assuming that discovery of known binders was a representative sampling from the pool of all binders the observed similarity further enforces reliability of our prediction.

In this work we revealed a thermodynamically selected DYNLL binding motif, which expanded our knowledge on the binding preference of the partner proteins. The comparative structural and functional analyses showed that in most interactions the phage-evolved consensus motif mimics natural binders but also utilizes a previously undetected binding capacity of DYNLL at the −5 position. Even considering only class A motifs we predicted about one hundred novel DYNLL partners and this way significantly widened the scope of the human interactome around DYNLL. This large number of binders is in good agreement with the proposed general dimerizing engine function of the DYNLL hub protein. We have already shown that the highest scored EML3 protein is indeed a tight binder of DYNLL. Experimental verification of binding capacity of a random sample of additional predicted motifs is underway in our laboratory.

Phage display has been an exceptionally powerful approach to characterize linear binding motif preference and in some cases predict natural binders of several peptide recognition modules including EH [54], PDZ [55], [56], [57], [58], [59], [60], PTB [61], SH3 [62], [63], [64] and WW domains [65].

In this study we comprehensively characterized the linear motif preference of the DYNLL hub protein and predicted novel binders. We hope that our DYNLL binder classes will provide a rich source of valuable information for research groups studying proteins listed in the predicted motif sets. The more of the predicted interactions are verified the better we will understand how DYNLL participates in organizing diverse protein networks and we will also learn more about how these networks function.

## Materials and Methods

All chemicals unless otherwise specified were purchased from Sigma-Aldrich. DNA modifying enzymes were from Fermentas and New England Biolabs.

### Expression vector constructions

For bacterial GST-fused expression the DYNLL1 gene was cloned into the pGEX4T-1 expression vector (Amersham) between *BamH*I and *Nde*I restriction sites using PCR. For His-tagged bacterial expression DYNLL1 and DYNLL2 (residues: 1–89, Uniprot accession numbers: P63167 and Q96FJ2, respectively) were cloned into the pET-15b (Novagen) expression vector as previously described [20]. The leucine zipper from yeast GCN4 peptide (residues: 250–281) was cloned between *BamH*I and *Bgl*II sites into a modified pET expression vector which allows N-terminal GST fusion and cleavage by TEV protease.

### Protein expression, purification and peptide synthesis

6xHis-tagged DYNLL1 and DYNLL2; the N-terminal GST-tagged GCN4 leucine zipper-fused DYNLL-binding peptides and the N-terminal GST-tagged DYNLL1 were expressed in *E. coli* BL21(DE3)Star cells (Novagen). The cells were allowed to grow in LB media up to OD_600_ = 1.5 at 37°C and induced overnight with 0.5 mM IPTG at 18°C. After affinity chromatography using GST-Bind™ Resin (Novagen) or Profinity™ IMAC Ni-Charged Resin (Bio-RAD), the N-terminal GST or 6xHis tags were removed by proteolysis. In case of the GCN4 leucine zipper-fused DYNLL-binding peptides TEV protease [66], while in case of DYNLL2, thrombin was used. The DYNLL1, DYNLL2 and GST-tagged DYNLL1 proteins were further purified by anion exchange chromatography using HiTrapQ ion-exchange column (Amersham) as previously described [30].

Short synthetic peptides were produced in-house using an ABI 431A Peptide Synthesizer and standard Fmoc chemistry. The N-terminus of the peptides was acetylated.

The GCN4 leucine zipper-fused peptides and the synthetic peptides were purified by RP-HPLC using Jupiter-300 5 µm C18 300 E 10×250 mm column (Phenomenex). Peptide and protein identities were confirmed by mass spectrometry. Protein concentrations were estimated by absorbance at 280 nm using calculated molar extinction coefficients; concentration of peptides was estimated by amino acid analysis.

### Phagemid vector construction

The pS1602a phagemid [67] displaying human growth hormone (hGH) on the p3 coat protein of the M13 phage under the control of a pTac promoter was generously provided by Genentech. This vector contains an NsiI site between the signal peptide and the hGH coding sequence. Between the hGH and the p3 protein coding segments there is a glycine-serine (G/S) linker coding segment, which does not contain unique restriction enzyme cleavage site. A unique KpnI site was introduced to the beginning of the G/S linker by Kunkel mutagenesis [68] resulting the phagemid, pG2B. Then, in several cloning steps using synthetic adaptors, primers and PCR reactions, the pTFBL-p3 vector was produced. This vector contains a pTac promoter, a malE signal peptide and a p3 coding segment from pS1602a [67]. Between the signal peptide and the p3 segment it contains a FLAG-tag (DYKDDDD), a 5 residue G/S linker, an 8 residue (EDKATQTL) DLC-binding peptide from the Bmf protein (residues: 66–73; Uniprot accession number Q96LC9) [5], a 4 residue G/S linker and a Leu-zipper from the yeast GCN4 protein (residues 250–281, Uniprot accession number: P03069). The Leu-zipper segment was obtained by a PCR reaction from yeast cDNA library [69]. In a subsequent step the pTac promoter was replaced with a PhoA promoter by replacing a cassette between the EcoRI and NsiI sites of the pTFBL-p3 vector with a cassette between the same sites from the phGHR(1–238) vector [42], which was a generous gift from Genentech. This final step resulted in the vector pPFBL-p3 which is illustrated in Figure 1A.

### Library construction

The library was produced based on previously described protocols [70]. In the pPFBL-p3 vector the Bmf octapeptide coding segment was replaced by 8 consecutive TAA stop codons by Kunkel mutagenesis [68] resulting in the pPF-STOP-L-p3 vector. This vector served as a stop template for Kunkel mutagenesis, when stop codons were replaced with the following segment: NNK NNK NNK NNK NNK CAG NNK NNK. The NNK codons represent a set of 32 codons covering all 20 amino acid residues. CAG codes for glutamine. The resulted pPF-Lib-p3 library construct is illustrated in Figure 1B and C.

### Phage display

All steps of the phage display selection and analysis of individual peptide-displaying clones were carried out as previously described [70]. At first DYNLL1 was immobilized onto MaxiSorp plates (NUNC), but the immobilization efficiency was poor. The amount of properly folded immobilized homodimer DYNLL1 was increased by using the GST-fused form. The increased yield might have been due to two factors. Larger proteins immobilize better on the MaxiSorp plate and GST is a homodimer, which as a tag, appears to stabilize the homodimer state of DYNLL1 [71]. To prevent selection of GST-binding peptides, the peptide-phage solution was supplemented with 30 µg/ml recombinant GST. Two independent selections on two different targets were performed. Nunc MaxiSorp ELISA plates were coated with GST-DYNLL1 (3 µg/ml) or anti-Flag tag antibody (2 µg/ml; SIGMA, F3165), respectively. Three selection rounds were carried out on each target separately as described. The eluted phage population was amplified in XL1 Blue cells superinfected with helper phage. Binding properties of individual peptide-phage clones were tested in a phage-ELISA format as described [70].

### Sequence analysis

The genes of individual peptide-phage clones producing an ELISA signal on their target 3-fold above background (measured on BSA or casein containing wells) were sequenced by the Big Dye Terminator v3.1 cycle Sequencing Kit (Applied Biosystems). To eliminate the effects of display bias, the amino acid frequencies determined for DYNLL1-binding peptide-phage population were normalized to data from the anti-Flag tag selected population. At each randomized position, the positional amino acid frequencies from the DYNLL-selected population were normalized to the positional frequencies of amino acids determined in the anti-Flag tag selected population. Sequences of the DYNLL-selected and the anti-Flag tag selected clones are listed in Table S2 and S3, respectively. For logo representation of the normalized results an input sequence dataset containing 100 sequences was generated representing the normalized amino acid frequencies at each randomized positions. Sequence logos were generated by the online application, WebLogo [44] available at http://weblogo.berkeley.edu/logo.cgi.

### Isothermal titration calorimetry

All peptides and DYNLL1 were dialyzed overnight in PBS buffer supplemented with 3 mM 2-mercaptoethanol (pH 7.4). A typical binding experiment involved 15 µM DYNLL1 in the cell and 30–40 injections varying between of 3–5 µl peptide solution. For each experiment the thermodynamic parameters were determined at 299 K. We used a VP-ITC instrument from Microcal and the data were fitted by the software package Origin 5.0 (OriginLab) using the simple A + B to AB binding model. The signal from an initial 2 µl injection was eliminated. The equilibration time between injections was 900 sec.

### Crystallographic studies

DYNLL2 was dialyzed twice against TBS (20 mM TRIS-HCl, 150 mM NaCl, 3 mM NaN_3_, 5 mM DTT, pH 7.6) and concentrated using Amicon Ultra-4 Centrifugal Filter Unit with Ultracel-3 membrane (Millipore). The Ac-SRGTQTE peptide and the dimerized-VSRGTQTE peptide were dissolved in the same buffer and subsequently complexed with DYNLL2. In the final solutions of the complexes the Ac-SRGTQTE concentration was 2.0 mM, the dimerized-VSRGTQTE concentration was 1.3 mM, while the concentration of dimeric DYNLL2 was 1.5 mM and 1.2 mM, respectively. Crystals were grown using the hanging drop method at 293 K with a reservoir solution of 31% PEG4000, 0.4 M CH_3_COONH_4_, 0.1 M CH_3_COONa pH 4.6 in the case of the Ac-SRGTQTE complex and 20% PEG8000, 0.2 M MgCl_2_, 0.1 M TRIS pH 7.0 in the case of the dimerized-VSRGTQTE complex. Drops were composed of 2 µl reservoir solution and 2 µl protein solution. The crystals were soaked in reservoir solution plus 20% glycerol for 1 minute and subsequently flash frozen in liquid nitrogen.

X-ray diffraction data were collected from a crystal of the Ac-SRGTQTE complex at 100 K with an ADSC Q315R CCD detector at ID29 of the ESRF (λ = 0.93 Å). Data were indexed, integrated and scaled to a resolution of 1.31 Å using XDS and XSCALE [72]. The space group is P2_1_2_1_2_1_ with unit cell dimensions of a = 35.6 Å, b = 64.0 Å, c = 151.8 Å. Data collection of the crystal of the dimerized GSVSRGTQTE complex was carried out using a Rigaku R-AXIS IV++ detector with Cu Kα radiation (λ = 1.5418 Å) focused by Osmic confocal optics. Data were indexed, integrated and scaled to 2.9 Å resolution using the CrystalClear software from Rigaku. The space group is P2_1_2_1_2_1_ with unit cell dimensions a = 53.8 Å, b = 68.4 Å, c = 101.7 Å.

The structure of the DYNLL2/Ac-SRGTQTE peptide complex was solved by molecular replacement using the program PHASER [73] of CCP4 6.1.2 Program Suite [74], [75]. As search model, the structure of 1CMI was used, thereafter automated model building was carried out with Arp/wArp [76] using amino acid sequences of both DYNLL2 monomer and the synthetic peptide Ac-SRGTQTE. The model was systematically improved using iterative cycles of manual rebuilding with the program Coot [77] and refinement with Refmac5 of CCP4 6.1.2 Program Suite [74], [75]. At the final steps of refinement restrained anisotropic temperature factors for the protein atoms were introduced and this step reduced R_factor_ and R_free_ by 2.4% and 1.5% respectively. During the automated model building, 419 water molecules were modeled and the final refined structure had overall R_cryst_ = 12.1% and R_free_ = 15.6%. The structure of the DYNLL2/Leu-zipper dimerized GSVSRGTQTE peptide complex was solved using the MOLREP program off the CCP4 package [78]. The DYNLL2/Ac-SRGTQTE complex structure was used as search model. Model building was carried out using the program Coot [77]. The model was refined with Refmac5 [75] using restrained maximum-likelihood refinement and TLS refinement [79]. During refinement non-crystallographic restraints were added to the DYNLL2 molecules of the dimer as well as to the bound segment and coiled coil region of the engineered peptide. The final model contains 1999 atoms and 40 water molecules and it has overall R_cryst_ = 25.0% and R_free_ = 29.5%. The stereochemistry of the structures was assessed with WHATCHECK [80] and PROCHECK [81]. The structures were deposited with the PDB under reference codes 2XQQ and 3P8M, respectively. Crystallographic data and refinement statistics are shown in Table S4.

### Binding partner prediction

For binding partner prediction a simple scoring matrix was used containing display-normalized position-specific percentages of each amino acid type of the selected sequence set. The score equals the summed up occurrences along the eight residue motif (Eq. 1).
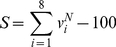
(1)


S is the score of the eight residue motif, 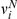 is the percentage occurrence of amino acid N in the i^th^ position in the selected sequence set. Subtraction of 100 stands for eliminating the score contribution of the nonrandomized Gln at position 0. The input dataset was a filtered subset of the annotated human protein sequence set from UniProt [82]. Secreted proteins, transmembrane and extracellular domains of membrane proteins were excluded. From the remaining intracellular set only predicted disordered regions were used. A score value was assigned to each eight residue segment if at least one residue of the segment was predictedly unstructured and if the segment contained a Gln at position 0. For disorder prediction we used IUPred [83] as proposed by a recent comparative study [84]. The prediction program was implemented in Perl and is freely available with the scoring matrix upon request. (For an overview of binding site prediction see Figure 5.)

We determined the threshold as follows: we generated 1000 scoring matrices by keeping the original phage selected matrix scores at each position but randomized the positions themselves. Using the same input sequence set, we repeated the above described scoring with all randomized matrices. To calculate the probability density function, hits were summed up for ten score unit windows. Density distribution function values generated by the real phage selected scoring matrix were normalized by the averages of corresponding values obtained by the randomized matrices. Threshold was defined as a score value above which the normalized distribution function values exceed one.

## Supporting Information

Figure S1 **Peptide display level.** (A) Effect of IPTG induction on the display level of the IPTG inducible pTac promoter driven pTBL-p3 construct. (B) Replacing of the pTac promoter with phoA promoter results in ∼30-fold increase in display level.
(DOC)

Figure S2 **ITC measurement of the (8–94) residues fragment of EML3 binding to DYNLL1.**
(DOC)

Table S1 **41 known DYNLL binding motifs from 33 proteins.**
(DOC)

Table S2 **25 non-identical (based on DNA level) phage selected sequences.**
(DOC)

Table S3 **30 non-identical (based on DNA level) Flag-tag selected sequences.**
(DOC)

Table S4 **Crystallographic data and refinement statistics.**
(DOC)

Table S5 **List of predicted DYNLL binding motifs with scores higher than the threshold level (≥220).** Sequences are sorted in three classes according to the reliability of the prediction: **A**) most probable, **C**) least probable interacting partners. For a detailed description of the classification rules see the main text.
(DOC)
