# Supplementary material for: Directed Evolution Reveals the Binding Motif Preference of the LC8/DYNLL Hub Protein and Predicts Large Numbers of Novel Binders in the Human Proteome
Source: PLoS One. 2011 Apr 18;6(4):e18818. doi: 10.1371/journal.pone.0018818 (PMC3078936; doi:10.1371/journal.pone.0018818)
Supplement: Table S1 — 41 known DYNLL binding motifs from 33 proteins. (DOC) [file pone.0018818.s003.doc]

| **Protein name*** | **Uniprot code** | **Sequence** | **Score** | **Residue** | **Multimerization**** |
| --- | --- | --- | --- | --- | --- |
| Adenain | P11826 | LVKSTQTV | 218 | 107 | - |
| Bassoon | Q9UPA5 | ANYGSQTE | 176 | 1426 | Predicted coiled coil |
| Bassoon | Q9UPA5 | VAQGTQTP | 269 | 1530 | Predicted coiled coil |
| Bassoon | Q9UPA5 | AEFSTQTP | 188 | 1502 | Predicted coiled coil |
| Bcl-2-like protein 11, BimL | O43521 | CDKSTQTP | 214 | 110 | - |
| Bcl-2-modifying factor, Bmf | Q96LC9 | EDKATQTL | 220 | 66 | - |
| Breast carcinoma-amplified sequence 1 | O75363 | LDAQVQTD | 104 | 566 | Homodimer |
| Cytoplasmic dynein 1 intermediate chain 1 | O88485 | YSKETQTP | 246 | 149 | Homodimer |
| DNA (cytosine-5)-methyltransferase 3A | Q9Y6K1 | KDLGIQVD | 70 | 651 | Homodimer |
| Human papillomavirus E4 | P06425 | QDKQTQTP | 202 | 21 | - |
| Egalitarian | P92030 | VDAESQTL | 135 | 950 | Predicted coiled coil |
| Guanylate kinase-associated protein, GKAP | O14490 | QSVGVQVE | 149 | 675 | Predicted coiled coil |
| Guanylate kinase-associated protein, GKAP | O14490 | LSIGIQVD | 122 | 650 | Predicted coiled coil |
| Microtubule-associated protein 4 | P27816 | GSKSTQTV | 259 | 800 | - |
| Myeloid leukemia factor 1, Mlf1 | P58340 | FQASTQTR | 189 | 124 | - |
| Myosin Va | Q9Y4I1 | DDKNTMTD | 108 | 1284 | Homodimer |
| Nitric oxide synthase, brain | P29475 | KDMGIQVD | 70 | 239 | Homodimer |
| Nuclear respiratory factor 1 | Q16656 | EHGVTQTE | 210 | 3 | Homodimer |
| Nucleoporin NUP159 | P40477 | ADFDVQTS | 107 | 1105 | Predicted coiled coil |
| Nucleoporin NUP159 | P40477 | AESGIQTD | 142 | 1118 | Predicted coiled coil |
| Nucleoporin NUP159 | P40477 | CNFSVQTF | 132 | 1167 | Predicted coiled coil |
| Nucleoporin NUP159 | P40477 | KHNSTQTV | 208 | 1143 | Predicted coiled coil |
| Nucleoporin NUP159 | P40477 | VDNGLQTE | 200 | 1155 | Predicted coiled coil |
| Lyssavirus phosphoprotein. | O56780 | EDKSTQTP | 214 | 142 | Homotrimer |
| Rabiesvirus P protein | P15198 | EDKSTQTT | 215 | 142 | Homotrimer |
| African swine fever virus p54 | Q4TWM2 | QNTASQTM | 130 | 142 | - |
| Swallow | P40688 | SAKATQTD | 231 | 289 | Homodimer |
| Ras guanyl-releasing protein 3 | Q8IV61 | TSQATQTE | 262 | 610 | homodimer |
| Replication origin-binding protein | P10193 | MAKSTQTF | 233 | 744 | Homodimer |
| Serine/threonine-protein kinase Nek9 | Q8TD19 | HSKGTQTA | 293 | 943 | Homodimer |
| Serine/threonine-protein kinase PAK 1 | Q13153 | RDVATSPI | 46 | 215 | Homodimer. |
| Tumor suppressor p53-binding protein 1 | Q12888 | NNIGIQTM | 177 | 1150 | Homooligomers |
| Tumor suppressor p53-binding protein 1 | Q12888 | VSAATQTI | 290 | 1167 | Homooligomers |
| Zinc finger MYND domain-containing protein 11 | Q15326 | LHRSTQTT | 239 | 411 | Predicted coiled coil |
| Zinc finger protein 354A | Q02975 | TTKSTQTQ | 229 | 94 | - |
| Gephyrin | Q03555 | EDKGVQCE | 98 | 219 | Homooligomers |
| Zinc finger transcription factor, Trps1 | Q9UHF7 | VDRSTQDE | 207 | 1205 | - |
| Heat shock cognate 71 kDa protein, Hsc73 | P63018 | PTKQTQTF | 221 | 430 | Predicted coiled coil |
| Guanine nucleotide-binding protein subunit beta-2-like 1, Rack1 | P63244 | CKYTVQDE | 30 | 138 | - |
| Zaire Ebola virus; Polymerase cofactor, VP35 | Q05127 | RNSQTQTD | 215 | 69 | Homooligomers |
| Syntaphilin | B5DF41 | QERAIQTD | 155 | 310 | Predicted coiled coil |

*: The background of motifs from human proteins is colored according to the assigned score: purple – motif below the threshold level, green – motif above the threshold level, orange – motif contains other residue than Gln at the 0^th^ position therefore has no assigned score (for details see the main text).

**: The UniProt sequence annotation and the COILS coiled coil predictor [1] were used to assign multimerizing capacity to the proteins. “Homodimers” contain experimentally verified α-helical coiled coils or associate via other motifs.

**Supporting Reference:**

1. Lupas A, Van Dyke M, Stock J (1991) Predicting coiled coils from protein sequences. Science 252: 1162-1164.
