# Supplementary material for: Directed Evolution Reveals the Binding Motif Preference of the LC8/DYNLL Hub Protein and Predicts Large Numbers of Novel Binders in the Human Proteome
Source: PLoS One. 2011 Apr 18;6(4):e18818. doi: 10.1371/journal.pone.0018818 (PMC3078936; doi:10.1371/journal.pone.0018818)
Supplement: Table S2 — 25 non-identical (based on DNA level) phage selected sequences. (DOC) [file pone.0018818.s004.doc]

| | # | Sequence | | --- | --- | | 1 | HSVAVQTE | | 2 | LSKGTQTT | | 3 | ISVGTQTD | | 4 | ITRGTQTG | | 5 | ITVSTQTE | | 6 | VSIGIQTT | | 7 | VSRATQTV | | 8 | VTKATQTS | | 9 | VTRATQTS | | 10 | VTRGTQTS | | 11 | VTRSTQTY | | 12 | VHVSTQTR | | 13 | MSRGTQTH | | 14 | MSRGTQTS | | 15 | NAKWTQSS | | 16 | NTRYTQTI | | 17 | RTIGTQTY | | 18 | RTVGTQTE | | 19 | RNAWTQTY | | 20 | RNVATQTP | | 21 | RSIATQTS | | 22 | RSIGIQVF | | 23 | RSVAVQTD | | 24 | RSVSTQTH | | 25 | KTVGTQTT | |  |
| --- | --- | --- | --- | --- | --- | --- | --- | --- | --- | --- | --- | --- | --- | --- | --- | --- | --- | --- | --- | --- | --- | --- | --- | --- | --- | --- | --- | --- | --- | --- | --- | --- | --- | --- | --- | --- | --- | --- | --- | --- | --- | --- | --- | --- | --- | --- | --- | --- | --- | --- | --- | --- | --- |
