# Supplementary material for: Directed Evolution Reveals the Binding Motif Preference of the LC8/DYNLL Hub Protein and Predicts Large Numbers of Novel Binders in the Human Proteome
Source: PLoS One. 2011 Apr 18;6(4):e18818. doi: 10.1371/journal.pone.0018818 (PMC3078936; doi:10.1371/journal.pone.0018818)
Supplement: Table S3 — 30 non-identical (based on DNA level) Flag-tag selected sequences. (DOC) [file pone.0018818.s005.doc]

| # | Sequence |
| --- | --- |
| 1 | PLSRVQGS |
| 2 | GVAVLQTE |
| 3 | TARLLQDH |
| 4 | FSDNKQTN |
| 5 | TTEFIQQD |
| 6 | QTTLVQNR |
| 7 | PVFYFQSK |
| 8 | YGISTQQL |
| 9 | CTTRGQCA |
| 10 | YMGGGQWA |
| 11 | IVTGSQQR |
| 12 | RTLYSQPL |
| 13 | YTHDIQAH |
| 14 | LFPSTQHI |
| 15 | SQVRQQLL |
| 16 | TDGLWQEW |
| 17 | FYPASQHA |
| 18 | ISHGFQLM |
| 19 | RATRAQAN |
| 20 | GWVLQQGQ |
| 21 | PTAVQQKQ |
| 22 | RRDQTQLR |
| 23 | ILSQWQEH |
| 24 | GLALGQQM |
| 25 | RQPPYQWL |
| 26 | FWTSEQRS |
| 27 | YVVTSQAN |
| 28 | TGYLCQLF |
| 29 | VAGRAQGP |
| 30 | NLWVNQWP |
