# Supplementary material for: Directed Evolution Reveals the Binding Motif Preference of the LC8/DYNLL Hub Protein and Predicts Large Numbers of Novel Binders in the Human Proteome
Source: PLoS One. 2011 Apr 18;6(4):e18818. doi: 10.1371/journal.pone.0018818 (PMC3078936; doi:10.1371/journal.pone.0018818)
Supplement: Table S4 — Crystallographic data and refinement statistics. (DOC) [file pone.0018818.s006.doc]

|  | DYNLL2 / Ac-SRGTQTE | DYNLL2 / Leu-zipper dimerized GSVSRGTQTE |
| --- | --- | --- |
| *Data collection* |  |  |
| Space group | P2_1_2_1_2_1_ | P2_1_2_1_2_1_ |
| Cell parameters  *a* (Å)  *b* (Å)  *c* (Å)              α,β,γ (°) | 35.6  64.0  151.8  90 | 53.8  68.4  101.7  90 |
| Resolution (Å) | 151.84-1.31 (1.34-1.31)^a^ | 42.31-2.90 (3.00-2.90) |
| Total reflections | 624141 | 26916 |
| Unique reflections | 81872 | 8273 |
| Completeness (%) | 96.0 (67.1) | 94.4 (96.6) |
| *R_sym_* (%)^b^ | 8.0 (39.3) | 13.1 (57.6) |
| <*I* / σ> | 15.7 (2.9) | 6.4 (1.5) |
| *Refinement* |  |  |
| Resolution (Å) | 151.84-1.31 | 42.31-2.90 |
| No. reflections | 77776 | 8271 |
| No. of amino acids  DYNLL2  Peptide | 347  24 | 174  85 |
| No. of modeled non-  hydrogen atom positions | 3625 | 2040 |
| Average *B*-factor  (all atoms, Å^2^) | 10.5 | 52.8 |
| *R_cryst_* (%) | 12.1 | 25.0 |
| *R_free_* (%)^c^ | 15.6 | 29.5 |
| R.m.s. deviation from  ideal bond length (Å) | 0.024 | 0.011 |
| R.m.s. deviation from  ideal bond angles (°) | 1.99 | 1.12 |
| Ramachandran plot  (% by PROCHECK)  Most favored  Additionally allowed  Generously allowed  Disallowed | 90.6  8.3  0.0  1.2 | 92.8  6.4  0.0  0.9 |
| PDB reference code | 2XQQ | 3P8M |

^a^Values in parentheses indicate statistics for the highest resolution shell.

^b^ *R_sym_* = Σ**_h_**Σ*_i_* |*I***_h_***_i_* - <*I***_h_**>| / Σ**_h_**Σ*_i_* <*I***_h_**>

^c^5.0% of the reflections in a test set for monitoring the refinement process.
