# Supplementary material for: Directed Evolution Reveals the Binding Motif Preference of the LC8/DYNLL Hub Protein and Predicts Large Numbers of Novel Binders in the Human Proteome
Source: PLoS One. 2011 Apr 18;6(4):e18818. doi: 10.1371/journal.pone.0018818 (PMC3078936; doi:10.1371/journal.pone.0018818)
Supplement: Table S5 — List of predicted DYNLL binding motifs with scores higher than the threshold level (≥220). Sequences are sorted in three classes according to the reliability of the prediction: A) most probable, C) least probable interacting partners. For a detailed description of the classification rules see the main text. (DOC) [file pone.0018818.s007.doc]

| **Class A)** All amino acids are represented in the phage selected set or in known natural motifs | | | | | | |
| --- | --- | --- | --- | --- | --- | --- |
| **#** | **UniProt ID** | **Prot name** | **Position** | **Sequences** | **Score** | **Dimeric nature*** |
| 1 | Q32P44 | EMAL3_HUMAN | 80 | VSRGTQTE | 367 | Predicted coiled coil |
| 2 | O43313 | ATMIN_HUMAN | 488 | VSRETQTS | 316 |  |
| 3 | Q9Y6D5 | BIG2_HUMAN | 621 | VSSGTQTT | 311 |  |
| 4 | Q9UPX6 | K1024_HUMAN | 471 | SSVGTQTE | 310 | Predicted coiled coil |
| 5 | O15061 | SYNEM_HUMAN | 1253 | ESVGTQTS | 301 | Predicted coiled coil |
| 6 | Q8TD19 | NEK9_HUMAN | 942 | HSKGTQTA | 293 | Homodimer |
| 7 | Q96QB1 | RHG07_HUMAN | 733 | VSNSTQTS | 293 |  |
| 8 | Q12888 | TP53B_HUMAN | 1166 | VSAATQTI | 290 |  |
| 9 | O94964 | CT117_HUMAN | 1137 | ASVGTQTI | 289 | Predicted coiled coil |
| 10 | Q9HAR2 | LPHN3_HUMAN | 1371 | VTTSTQTE | 275 | Predicted coiled coil |
| 11 | Q6DT37 | MRCKG_HUMAN | 712 | RNVGTQTL | 275 | Predicted coiled coil |
| 12 | A0JNW5 | UH1BL_HUMAN | 1394 | VTQATQTS | 274 | Predicted coiled coil |
| 13 | Q9BWV3 | CDAC1_HUMAN | 16 | RSVSTQTG | 273 |  |
| 14 | P46013 | KI67_HUMAN | 2618 | VERLTQTS | 272 |  |
| 15 | Q9UPQ7 | PZRN3_HUMAN | 353 | VDTGTQTD | 272 | Predicted coiled coil |
| 16 | Q9UPA5 | BSN_HUMAN | 1529 | VAQGTQTP | 269 | Predicted coiled coil |
| 17 | Q7Z4T9 | AAT1_HUMAN | 186 | STVGTQTD | 267 |  |
| 18 | Q9UBY0 | SL9A2_HUMAN | 752 | REKGTQTS | 266 |  |
| 19 | Q8N7K9 | YS059_HUMAN | 315 | LSSGTQTT | 266 |  |
| 20 | Q96RI0 | PAR4_HUMAN | 15 | LSGGTQTP | 265 |  |
| 21 | Q8IV61 | GRP3_HUMAN | 609 | TSQATQTE | 262 |  |
| 22 | Q9C0C7 | AMRA1_HUMAN | 1099 | TSQGTQTL | 260 |  |
| 23 | O43313 | ATMIN_HUMAN | 806 | SSVETQTS | 259 |  |
| 24 | Q8NEL9 | DDHD1_HUMAN | 793 | TTVGTQTL | 259 | Predicted coiled coil |
| 25 | P27816 | MAP4_HUMAN | 799 | GSKSTQTV | 259 |  |
| 26 | Q96BW5 | PTER_HUMAN | 102 | ISRDTQTL | 258 |  |
| 27 | Q9H4H8 | FA83D_HUMAN | 404 | SEVGTQTS | 257 |  |
| 28 | O95267 | GRP1_HUMAN | 668 | AHKATQTE | 256 | Predicted coiled coil |
| 29 | Q9HCD6 | TANC2_HUMAN | 1422 | VSIGLQTE | 256 |  |
| 30 | Q4VXU2 | PAP1L_HUMAN | 481 | ANIGTQTT | 255 |  |
| 31 | O94964 | CT117_HUMAN | 1147 | VSVGLQTD | 254 | Predicted coiled coil |
| 32 | Q9NR71 | ASAH2_HUMAN | 77 | SSTATQTS | 253 |  |
| 33 | A6NCI8 | CB078_HUMAN | 315 | SSRNTQTL | 253 |  |
| 34 | Q6P1L5 | F117B_HUMAN | 245 | RDKATQTE | 253 |  |
| 35 | Q96T58 | MINT_HUMAN | 3390 | EAKGTQTG | 253 | Predicted coiled coil |
| 36 | Q96CK0 | ZN653_HUMAN | 361 | VAAYTQTE | 253 |  |
| 37 | Q9BYP7 | WNK3_HUMAN | 850 | NSTSTQTS | 252 |  |
| 38 | Q8NA54 | IQUB_HUMAN | 265 | HNAGTQTV | 251 |  |
| 39 | O75161 | NPHP4_HUMAN | 579 | IVVGTQTR | 249 |  |
| 40 | O14576 | DC1I1_HUMAN | 165 | YSKETQTP | 246 | Predicted coiled coil |
| 41 | Q02962 | PAX2_HUMAN | 317 | NVSGTQTY | 246 |  |
| 42 | P0C6A0 | ZGLP1_HUMAN | 97 | DSKDTQTR | 246 |  |
| 43 | P15924 | DESP_HUMAN | 1948 | SHRETQTE | 245 | Predicted coiled coil |
| 44 | Q9Y4B5 | K0802_HUMAN | 1624 | RTMGTQTV | 245 | Predicted coiled coil |
| 45 | Q5VWX1 | KHDR2_HUMAN | 289 | NSYATQTQ | 245 |  |
| 46 | P78364 | PHC1_HUMAN | 322 | VSQGSQTE | 244 | Homodimer |
| 47 | Q8TDF6 | GRP4_HUMAN | 637 | RHAWTQTE | 243 |  |
| 48 | Q14149 | MORC3_HUMAN | 635 | NTAATQTE | 243 | Homodimer |
| 49 | Q96LW1 | Z354B_HUMAN | 92 | MTKSTQTQ | 243 |  |
| 50 | P17844 | DDX5_HUMAN | 549 | VSAGIQTS | 242 |  |
| 51 | Q8NDV7 | TNR6A_HUMAN | 753 | GSSATQTF | 242 | Predicted coiled coil |
| 52 | Q8N5S3 | CB073_HUMAN | 259 | TSGATQTT | 241 |  |
| 53 | Q6P1L5 | F117B_HUMAN | 389 | RSIDTQTP | 241 |  |
| 54 | Q86VQ1 | GLCI1_HUMAN | 343 | RSIDTQTP | 241 | Predicted coiled coil |
| 55 | O15060 | ZBT39_HUMAN | 232 | VSTGIQTS | 241 |  |
| 56 | Q00013 | EM55_HUMAN | 402 | TDQGTQTE | 240 | Heterodimer |
| 57 | Q9Y4F5 | K0284_HUMAN | 1278 | TSTATQTP | 240 | Predicted coiled coil |
| 58 | P37198 | NUP62_HUMAN | 59 | FSLATQTP | 240 | Predicted coiled coil |
| 59 | Q3KR37 | GRM1B_HUMAN | 597 | VAGSTQTR | 239 |  |
| 60 | P30305 | MPIP2_HUMAN | 51 | VTTLTQTM | 239 |  |
| 61 | Q15326 | ZMY11_HUMAN | 410 | LHRSTQTT | 239 | Predicted coiled coil |
| 62 | P29375 | KDM5A_HUMAN | 196 | LSTDTQTS | 236 | Predicted coiled coil |
| 63 | Q99996 | AKAP9_HUMAN | 1881 | HAKVTQTE | 234 | Predicted coiled coil |
| 64 | O43313 | ATMIN_HUMAN | 644 | SNIQTQTE | 234 |  |
| 65 | Q12830 | BPTF_HUMAN | 2522 | VQSSTQTL | 234 | Predicted coiled coil |
| 66 | Q68DE3 | K2018_HUMAN | 644 | ASNSTQTF | 234 | Predicted coiled coil |
| 67 | Q68DE3 | K2018_HUMAN | 1526 | LVQGTQTS | 234 | Predicted coiled coil |
| 68 | Q9H195 | MUC3B_HUMAN | 163 | SATGTQTS | 234 |  |
| 69 | Q8IX01 | SFR14_HUMAN | 695 | ATTGTQTL | 233 |  |
| 70 | Q5JSH3 | WDR44_HUMAN | 153 | TTKLTQTS | 232 |  |
| 71 | Q2M3A8 | CK036_HUMAN | 6 | ITSATQTS | 231 |  |
| 72 | Q9ULV3 | CIZ1_HUMAN | 424 | KQVQTQTY | 230 | Predicted coiled coil |
| 73 | Q9UBG3 | CRNN_HUMAN | 310 | GSTSTQTQ | 230 | Homodimer |
| 74 | Q9UIG5 | PS1C1_HUMAN | 11 | RALGTQTP | 230 |  |
| 75 | Q6ZNE9 | RUFY4_HUMAN | 179 | CSSSTQTQ | 230 | Predicted coiled coil |
| 76 | Q86VQ1 | GLCI1_HUMAN | 197 | KDKATQTP | 229 | Predicted coiled coil |
| 77 | P14314 | GLU2B_HUMAN | 256 | LSGDTQTD | 229 | Heterodimer |
| 78 | Q8N7K9 | YS059_HUMAN | 393 | LSSETQTG | 229 |  |
| 79 | O60765 | Z354A_HUMAN | 92 | TTKSTQTQ | 229 |  |
| 80 | Q14202 | ZMYM3_HUMAN | 850 | KSKGSQTE | 229 |  |
| 81 | Q9Y4B5 | K0802_HUMAN | 1634 | ISVGLQTE | 227 | Predicted coiled coil |
| 82 | Q7Z6E9 | RBBP6_HUMAN | 615 | VSSGVQTA | 226 |  |
| 83 | P61577 | REC15_HUMAN | 65 | NTKVTQTP | 226 | Homodimer |
| 84 | P61579 | REC17_HUMAN | 65 | NTKVTQTP | 226 | Homodimer |
| 85 | P61571 | REC1_HUMAN | 64 | NTKVTQTP | 226 | Homodimer |
| 86 | Q69383 | REC2_HUMAN | 65 | NTKVTQTP | 226 | Homodimer |
| 87 | P61572 | REC3_HUMAN | 65 | NTKVTQTP | 226 | Homodimer |
| 88 | P61573 | REC4_HUMAN | 65 | NTKVTQTP | 226 | Homodimer |
| 89 | P61574 | REC5_HUMAN | 65 | NTKVTQTP | 226 | Homodimer |
| 90 | P61575 | REC6_HUMAN | 65 | NTKVTQTP | 226 | Homodimer |
| 91 | P61576 | REC9_HUMAN | 65 | NTKVTQTP | 226 | Homodimer |
| 92 | A7KAX9 | RHG32_HUMAN | 1003 | ASGQTQTG | 226 |  |
| 93 | O43313 | ATMIN_HUMAN | 760 | NSTETQTM | 225 |  |
| 94 | Q8TDM6 | DLG5_HUMAN | 837 | HNNSTQTD | 225 | Predicted coiled coil |
| 95 | Q9H4H8 | FA83D_HUMAN | 386 | IDAATQTE | 224 |  |
| 96 | Q15434 | RBMS2_HUMAN | 289 | YQRVTQTS | 224 |  |
| 97 | Q9H5J0 | ZBTB3_HUMAN | 198 | TSRGTQPS | 224 |  |
| 98 | Q9C0C7 | AMRA1_HUMAN | 1111 | QNAETQTE | 223 |  |
| 99 | Q92817 | EVPL_HUMAN | 1670 | LSQETQTR | 223 | Predicted coiled coil |
| 100 | Q14157 | UBP2L_HUMAN | 592 | EQRSTQTR | 223 |  |
| 101 | Q2T9L4 | CO059_HUMAN | 263 | RNSSTQTV | 222 | Predicted coiled coil |
| 102 | Q8TEC5 | SH3R2_HUMAN | 717 | TASGTQTV | 222 |  |
| 103 | Q7Z6B0 | CCD91_HUMAN | 17 | GSGETQTT | 221 | Homodimer |
| 104 | Q5TG30 | RHG40_HUMAN | 89 | LSTLTQTQ | 221 | Predicted coiled coil |
| 105 | Q8IXF9 | AQ12A_HUMAN | 267 | ASGDTQTP | 220 |  |
| 106 | A6NM10 | AQ12B_HUMAN | 267 | ASGDTQTP | 220 |  |
| 107 | Q96LC9 | BMF_HUMAN | 65 | EDKATQTL | 220 |  |
| 108 | Q6PJG2 | CN043_HUMAN | 966 | AVKATQTL | 220 |  |
| 109 | Q03060 | CREM_HUMAN | 39 | AHVQTQTG | 220 | Homodimer |
| 110 | Q5VSD8 | YI029_HUMAN | 66 | KNTSTQTT | 220 |  |

| **Class B)** Residues (up to three) missing from the phage selected set or from known natural motifs | | | | | | |
| --- | --- | --- | --- | --- | --- | --- |
| **#** | **UniProt ID** | **Prot name** | **Position** | **Sequences**** | **Score** | **Dimeric nature*** |
| 1 | Q9Y2G4 | ANKR6_HUMAN | 613 | VNRGTQTK | 323 | Predicted coiled coil |
| 2 | Q15326 | ZMY11_HUMAN | 393 | VSVSTQTK | 304 | Predicted coiled coil |
| 3 | Q8NCP5 | ZBT44_HUMAN | 195 | VKCGTQTS | 279 |  |
| 4 | Q99550 | MPP9_HUMAN | 756 | KNWGTQTE | 271 | Predicted coiled coil |
| 5 | Q9Y228 | T3JAM_HUMAN | 161 | HHRGTQTK | 270 | Predicted coiled coil |
| 6 | Q03164 | MLL1_HUMAN | 3479 | VSNFTQTV | 269 | Homo-, heterodimer |
| 7 | Q8NDZ9 | YJ017_HUMAN | 196 | GLRGTQTS | 266 |  |
| 8 | P46013 | KI67_HUMAN | 1648 | VGKLTQTS | 263 |  |
| 9 | P46013 | KI67_HUMAN | 2014 | VGKLTQTS | 263 |  |
| 10 | P46013 | KI67_HUMAN | 2857 | VGKLTQTS | 263 |  |
| 11 | A4GXA9 | EME2_HUMAN | 194 | VSRGTQQP | 259 |  |
| 12 | Q15528 | MED22_HUMAN | 47 | VSRATQGE | 259 |  |
| 13 | P51659 | DHB4_HUMAN | 196 | GSRMTQTV | 256 |  |
| 14 | Q9HAU0 | PKHA5_HUMAN | 1076 | VSRGNQTM | 255 | Predicted coiled coil |
| 15 | Q8TBR5 | CS023_HUMAN | 25 | RSVLTQTK | 253 |  |
| 16 | Q8N6M8 | IQCF1_HUMAN | 40 | VLVETQTV | 251 |  |
| 17 | Q5VUA4 | ZN318_HUMAN | 1339 | VTTSTQTK | 251 | Predicted coiled coil |
| 18 | Q96FN5 | KIF12_HUMAN | 562 | HSDWTQTR | 250 | Predicted coiled coil |
| 19 | O75369 | FLNB_HUMAN | 1604 | RIRATQTG | 246 | Homodimer |
| 20 | Q86UW6 | N4BP2_HUMAN | 1010 | VGMCTQTE | 246 |  |
| 21 | Q86UW6 | N4BP2_HUMAN | 1010 | VGMCTQTE | 246 |  |
| 22 | Q9BVV6 | K0586_HUMAN | 675 | KSIRTQTD | 245 | Predicted coiled coil |
| 23 | Q66K89 | E4F1_HUMAN | 658 | IIEGTQTE | 245 | Homodimer |
| 24 | P51788 | CLCN2_HUMAN | 650 | ERRATQTS | 244 |  |
| 25 | Q9H3D4 | P63_HUMAN | 39 | MSQSTQTN | 244 |  |
| 26 | P20930 | FILA_HUMAN | 2626 | GTRHTQTS | 241 | Predicted coiled coil |
| 27 | P20930 | FILA_HUMAN | 2950 | GTRHTQTS | 241 | Predicted coiled coil |
| 28 | P14859 | PO2F1_HUMAN | 16 | GNTGTQTN | 240 |  |
| 29 | Q9Y266 | NUDC_HUMAN | 170 | NYRWTQTL | 239 |  |
| 30 | Q9UPQ9 | TNR6B_HUMAN | 653 | ESAATQTK | 239 |  |
| 31 | Q01484 | ANK2_HUMAN | 2861 | SSITTQTD | 238 |  |
| 32 | Q9ULM2 | ZN490_HUMAN | 47 | QSIKTQTD | 238 |  |
| 33 | Q9H4H8 | FA83D_HUMAN | 437 | RSTTTQTD | 235 |  |
| 34 | P43378 | PTN9_HUMAN | 348 | RSGHTQTD | 235 |  |
| 35 | Q12830 | BPTF_HUMAN | 1634 | ESDSTQTT | 233 | Predicted coiled coil |
| 36 | Q9C091 | GRB1L_HUMAN | 270 | KSGFTQTD | 233 |  |
| 37 | Q01974 | ROR2_HUMAN | 763 | ASNTTQTS | 233 | Homodimer |
| 38 | Q9ULE0 | WWC3_HUMAN | 821 | CSNCTQTS | 233 | Predicted coiled coil |
| 39 | Q5JPB2 | ZN831_HUMAN | 188 | KHRRTQTH | 233 | Predicted coiled coil |
| 40 | Q9H320 | VCX1_HUMAN | 94 | VSEGTQHD | 230 |  |
| 41 | Q9H322 | VCX2_HUMAN | 94 | VSEGTQHD | 230 |  |
| 42 | Q9H321 | VCX3B_HUMAN | 94 | VSEGTQHD | 230 |  |
| 43 | Q9NNX9 | VCX3_HUMAN | 94 | VSEGTQHD | 230 |  |
| 44 | O14598 | VCY1_HUMAN | 94 | VSEGTQHD | 230 |  |
| 45 | Q9UQN3 | CHM2B_HUMAN | 82 | TSMSTQTK | 230 | Multimer |
| 46 | Q68CZ2 | TENS3_HUMAN | 581 | SSYSTQTW | 230 |  |
| 47 | P21333 | FLNA_HUMAN | 1501 | NADGTQTV | 229 | Homodimer |
| 48 | Q8IWJ2 | GCC2_HUMAN | 1320 | KSEHTQTV | 228 | Homodimer |
| 49 | Q9NYV4 | CDK12_HUMAN | 606 | VSVKTQVS | 228 |  |
| 50 | Q8N7Y1 | PRR10_HUMAN | 137 | RSWVTQTL | 227 |  |
| 51 | Q5T6F2 | UBAP2_HUMAN | 1018 | VYNKTQTF | 226 |  |
| 52 | Q5T7W0 | ZN618_HUMAN | 310 | VAAKTQTN | 226 |  |
| 53 | Q16625 | OCLN_HUMAN | 300 | VSAGTQDV | 226 | Predicted coiled coil |
| 54 | P61578 | REC16_HUMAN | 65 | NTKMTQTP | 226 | Homodimer |
| 55 | Q96L96 | ALPK3_HUMAN | 824 | RGDGTQTA | 225 |  |
| 56 | Q9Y6J0 | CABIN_HUMAN | 21 | KSHKTQTK | 225 |  |
| 57 | Q00975 | CAC1B_HUMAN | 1936 | VSWGTQRT | 225 | Predicted coiled coil |
| 58 | Q8NEP4 | CQ047_HUMAN | 104 | KSQKTQTL | 225 |  |
| 59 | Q9Y6Q9 | NCOA3_HUMAN | 1019 | VSHGTQNR | 224 |  |
| 60 | O15530 | PDPK1_HUMAN | 26 | SMVRTQTE | 224 |  |
| 61 | Q68CP9 | ARID2_HUMAN | 1201 | TMSGTQTG | 224 |  |
| 62 | Q6ZWK4 | CA186_HUMAN | 66 | EMKETQTE | 224 |  |
| 63 | Q96MH7 | CE034_HUMAN | 535 | CRRLTQTS | 224 |  |
| 64 | Q5VTT5 | MYOM3_HUMAN | 609 | AFRDTQTS | 224 |  |
| 65 | Q6KC79 | NIPBL_HUMAN | 641 | TKVETQTE | 224 | Predicted coiled coil |
| 66 | Q99952 | PTN18_HUMAN | 365 | AGSGTQTG | 224 |  |
| 67 | Q7Z4V0 | ZN438_HUMAN | 684 | GSKGTQEE | 224 |  |
| 68 | Q96K76 | UBP47_HUMAN | 523 | VSRITQED | 223 |  |
| 69 | Q9H819 | DJC18_HUMAN | 50 | ENEWTQTR | 223 |  |
| 70 | Q14966 | ZN638_HUMAN | 1250 | ISGITQTM | 223 | Predicted coiled coil |
| 71 | Q96G01 | BICD1_HUMAN | 848 | VSSGTQRK | 222 | Predicted coiled coil |
| 72 | Q3L8U1 | CHD9_HUMAN | 1584 | KKVKTQTS | 222 |  |
| 73 | Q9P1V8 | CN174_HUMAN | 390 | VEEKTQTK | 222 |  |
| 74 | Q9BYB4 | GNB1L_HUMAN | 14 | VLRGTQSP | 222 |  |
| 75 | Q6Q0C1 | HDMCP_HUMAN | 120 | VRLQTQTQ | 222 |  |
| 76 | P51957 | NEK4_HUMAN | 708 | VQLMTQTL | 222 |  |
| 77 | Q01804 | OTUD4_HUMAN | 613 | VLSVTQTL | 222 |  |
| 78 | Q7Z7L9 | ZSCA2_HUMAN | 124 | VEDLTQTL | 222 |  |
| 79 | P49796 | RGS3_HUMAN | 116 | RDEWTQTS | 221 | Heterodimer |
| 80 | A4IF30 | S35F4_HUMAN | 132 | ADDGTQTH | 221 |  |
| 81 | Q9ULH7 | MKL2_HUMAN | 653 | VSTGGQTL | 220 | Predicted coiled coil |
| 82 | Q8WXR4 | MYO3B_HUMAN | 1173 | NNGRTQTS | 220 |  |
| 83 | Q86SE5 | RALYL_HUMAN | 0 | MTGKTQTS | 220 | Predicted coiled coil |
| 84 | O95785 | WIZ_HUMAN | 210 | SEVATQTW | 220 | Heterodimer |

| **Class C**) Prolines present between -5^th^ – 0^th^ positions of the binding motif | | | | | | |
| --- | --- | --- | --- | --- | --- | --- |
| # | **UniProt ID** | **Prot name** | **Position** | **Sequences**** | **Score** | **Dimeric nature*** |
| 1 | Q7Z591 | AKNA_HUMAN | 933 | VSPLTQTP | 268 | Predicted coiled coil |
| 2 | Q96T58 | MINT_HUMAN | 3444 | VSLPTQTA | 266 | Predicted coiled coil |
| 3 | Q9Y2X9 | ZN281_HUMAN | 649 | LSPGTQTP | 265 |  |
| 4 | P01133 | EGF_HUMAN | 1167 | PSYGTQTL | 260 |  |
| 5 | Q2M1K9 | ZN423_HUMAN | 1152 | PRKGTQTS | 257 | Homodimer |
| 6 | Q9ULV3 | CIZ1_HUMAN | 288 | VPKQTQTP | 250 | Predicted coiled coil |
| 7 | Q8IX15 | HOMEZ_HUMAN | 147 | LSKPTQTK | 247 | Homo-, heterodimer |
| 8 | Q6P1L5 | F117B_HUMAN | 106 | PTVATQTG | 245 |  |
| 9 | Q9Y3S1 | WNK2_HUMAN | 1080 | QSVPTQTA | 244 | Predicted coiled coil |
| 10 | Q9Y5P3 | RAI2_HUMAN | 274 | PFKGTQTP | 244 |  |
| 11 | Q92793 | CBP_HUMAN | 905 | VPSATQTQ | 242 | Predicted coiled coil |
| 12 | Q9UPN3 | MACF1_HUMAN | 3412 | EPVGTQTA | 242 | Predicted coiled coil |
| 13 | Q96PK2 | MACF4_HUMAN | 3914 | EPVGTQTA | 242 | Predicted coiled coil |
| 14 | Q6ZT07 | TBCD9_HUMAN | 455 | VPTATQTL | 242 |  |
| 15 | Q9UPZ9 | ICK_HUMAN | 464 | NSAPTQTS | 241 |  |
| 16 | Q8NEZ4 | MLL3_HUMAN | 3493 | NSPSTQTF | 241 | Predicted coiled coil |
| 17 | Q8NAA6 | CO053_HUMAN | 170 | VTFPTQTR | 241 |  |
| 18 | Q14686 | NCOA6_HUMAN | 1199 | VAAPTQTS | 241 | Homodimer |
| 19 | P49746 | TSP3_HUMAN | 603 | MSNPTQTD | 240 |  |
| 20 | A5YM69 | ARG35_HUMAN | 430 | QIPGTQTE | 240 |  |
| 21 | Q12774 | ARHG5_HUMAN | 430 | QIPGTQTE | 240 |  |
| 22 | Q96GM8 | TOE1_HUMAN | 350 | NLPGTQTS | 238 |  |
| 23 | Q8N8K9 | K1958_HUMAN | 89 | VPSETQTS | 237 |  |
| 24 | P50542 | PEX5_HUMAN | 74 | VSRAPQTF | 237 |  |
| 25 | Q03164 | MLL1_HUMAN | 3427 | VLPSTQTT | 237 | Homo-, heterodimer |
| 26 | P57723 | PCBP4_HUMAN | 234 | LDPGTQTS | 234 |  |
| 27 | Q8N554 | ZN276_HUMAN | 575 | VHPLTQTQ | 234 |  |
| 28 | Q9H2F5 | EPC1_HUMAN | 721 | PSNITQTS | 233 |  |
| 29 | Q8N7K9 | YS059_HUMAN | 303 | PSSRTQTS | 233 |  |
| 30 | O60741 | HCN1_HUMAN | 680 | PSPSTQTP | 232 |  |
| 31 | O14686 | MLL2_HUMAN | 3968 | QEPGTQTS | 231 | Predicted coiled coil |
| 32 | Q8N187 | AL2S8_HUMAN | 116 | VIPPTQTG | 230 |  |
| 33 | Q14679 | TTLL4_HUMAN | 1161 | PSLSTQTL | 230 |  |
| 34 | Q96EY1 | DNJA3_HUMAN | 375 | IPPGTQTD | 229 |  |
| 35 | Q9H3P2 | NELFA_HUMAN | 402 | VAPTTQTP | 227 |  |
| 36 | Q9NS68 | TNR19_HUMAN | 405 | IHPATQTS | 226 |  |
| 37 | Q8NFA0 | UBP32_HUMAN | 1005 | ASSPTQTD | 226 |  |
| 38 | Q15018 | F175B_HUMAN | 405 | DPRNTQTS | 224 |  |
| 39 | O00555 | CAC1A_HUMAN | 1148 | NPSGTQTN | 223 |  |
| 40 | P78347 | GTF2I_HUMAN | 700 | VRTPTQTN | 222 | Potential homodimer |
| 41 | Q70CQ4 | UBP31_HUMAN | 430 | LSSPTQTA | 221 |  |
| 42 | Q4AC94 | C2CD3_HUMAN | 1910 | LSPQTQTA | 221 |  |
| 43 | Q0VF49 | K2012_HUMAN | 33 | TSPLTQTT | 221 | Predicted coiled coil |
| 44 | Q8NEN9 | PDZD8_HUMAN | 577 | VSKPTQGS | 221 | Predicted coiled coil |
| 45 | P54652 | HSP72_HUMAN | 423 | PTKQTQTF | 221 |  |
| 46 | P17066 | HSP76_HUMAN | 422 | PTKQTQTF | 221 |  |
| 47 | P11142 | HSP7C_HUMAN | 420 | PTKQTQTF | 221 |  |
| 48 | Q16204 | CCDC6_HUMAN | 429 | PSPNTQTP | 220 | Predicted coiled coil |
| 49 | A6NFN3 | FOX1C_HUMAN | 65 | PIAGTQTV | 220 |  |

* The UniProt sequence annotation and the COILS coiled coil predictor [1] were used to assign multimerizing capacity to the proteins. “Homodimers” contain experimentally verified α-helical coiled coils or associate via other motifs.

** Amino acid residues not represented in the phage-evolved set are colored red.

**Supporting Reference:**

1. Lupas A, Van Dyke M, Stock J (1991) Predicting coiled coils from protein sequences. Science 252: 1162-1164.
