# Supplementary material for: Directed Evolution Reveals the Binding Motif Preference of the LC8/DYNLL Hub Protein and Predicts Large Numbers of Novel Binders in the Human Proteome
Source: PLoS One. 2011 Apr 18;6(4):e18818. doi: 10.1371/journal.pone.0018818 (PMC3078936; doi:10.1371/journal.pone.0018818)
Supplement: Supplementary file 7 [file pone.0018818.s002.doc]

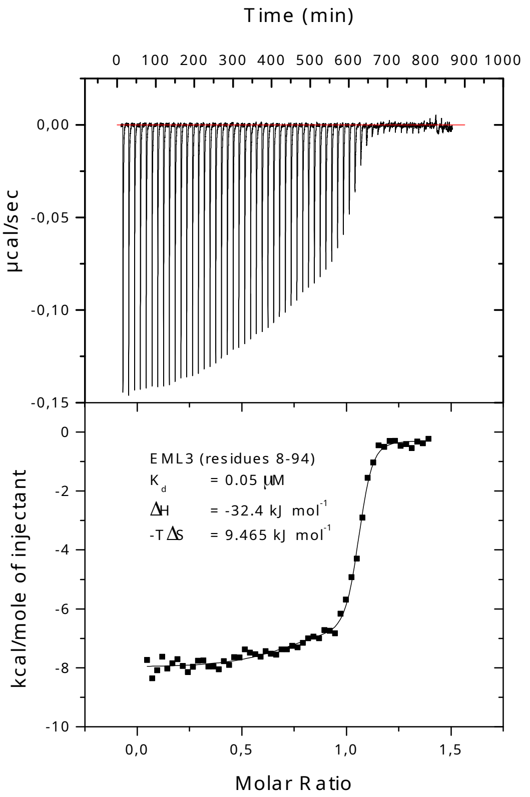

Time (min)
0 100 200 300 400 500 600 700 800 900 1000
µcal/sec
0,00
-0,05
-0,10
-0,15
kcal/mole of injectant
0
-2
-4
-6
-8
-10
EML3 (residues 8-94)
K_d = 0.05 µM
ΔH = -32.4 kJ mol^{-1}
-TΔS = 9.465 kJ mol^{-1}
0,0 0,5 1,0 1,5
Molar Ratio
